# Professional quality of life is related to emotional intelligence, self-care, and work conditions in healthcare workers: findings from a moderated mediation analysis

**DOI:** 10.1186/s12913-025-13437-7

**Published:** 2025-10-21

**Authors:** Lourdes Ferrer Goodwin, Akhtar Wallymahmed, Zoi Triandafilidis, Daniel Barker

**Affiliations:** 1 Brunel University London, Kingston Lane, London, Uxbridge, UB8 3PH UK; 2 Central Coast Research Institute, 77A Holden Street, Gosford, NSW 2113 Australia; 3 University of Newcastle, 77A Holden Street, Newcastle, NSW 2113 Australia; 4 Central Coast Local Health District, 77A Holden Street, Gosford, NSW 2113 Australia; 5 Hunter Medical Research Institute, Lot 1 Kookaburra Circuit, New Lambton Heights, NSW 2113 Australia

**Keywords:** Professional quality of life, Emotional intelligence, Self-care, Workplace social support, Type of work, Burnout, Compassion fatigue, Compassion satisfaction, Professional self-care, Personal self-care

## Abstract

**Background:**

Professional Quality of Life (ProQOL) among healthcare workers encompasses both positive aspects such as compassion satisfaction and negative dimensions including burnout and compassion fatigue. Emotional intelligence, self-care, workplace social support, and occupational role are critical factors influencing ProQOL outcomes, yet their interrelationships require further investigation within ecological frameworks.

**Objective:**

This study examined the direct and indirect effects of emotional intelligence on ProQOL in healthcare workers, with self-care as a mediator, and workplace social support and type of work as moderators, grounded in Bronfenbrenner’s Ecological Systems Theory, the Theory of Salutogenesis, and the Job Demands-Resources model.

**Methods:**

A cross-sectional survey of 343 healthcare workers from a single health district was conducted in New South Wales, Australia. Measures included emotional intelligence, personal and professional self-care (perception and practice), workplace social support, type of work, and ProQOL components. Data were analysed using SPSS and Process MACRO.

**Results:**

Emotional intelligence positively influenced compassion satisfaction, reduced burnout, and mitigated compassion fatigue both directly and indirectly via self‑care. Notably, professional self‑care was a stronger buffer against work‑related stress than personal self‑care. Effects varied by work type and workplace social support: stronger protective effects were observed among direct care providers, whereas non‑direct care providers showed attenuated or reversed associations for high emotional intelligence. Component‑specific effects were identified, with “self‑focus” elements emotional intelligence components linked to higher compassion fatigue, “use of emotions” to greater compassion satisfaction, and “regulation of emotions” to reduced burnout.

**Conclusions:**

Findings highlight the importance of targeted, context-sensitive interventions that strengthen professional self-care strategies, develop role-relevant emotional intelligence skills, and optimise workplace social support. Such approaches have the potential to enhance the professional quality of life of healthcare workers across diverse roles and organisational settings. Limitations of findings and recommendations for research and practice are discussed.

**Supplementary Information:**

The online version contains supplementary material available at 10.1186/s12913-025-13437-7.

## Background

### Healthcare workers ProQOL: importance and challenges

Professional Quality of Life (ProQOL) is a nuanced and multidimensional construct widely used to understand the psychological experiences of healthcare workers related to their caregiving roles. It addresses both the rewarding and taxing facets of care, encompassing three empirically distinct yet interrelated components: compassion satisfaction, burnout, and compassion fatigue [1]. Compassion satisfaction denotes the pleasure and fulfilment derived from effective caregiving and making a meaningful difference. It serves as a crucial protective factor that promotes resilience, professional engagement, and sustained empathy in healthcare settings [2–4].

Compassion fatigue, in contrast, which includes secondary traumatic stress, arises from sustained empathic engagement with individuals experiencing trauma and suffering, leading to emotional depletion, withdrawal, and trauma-specific symptoms [5–9]. Compassion Fatigue has been associated with mental health decline, professional performance and the quality of patient care [10, 11].

Burnout is characterised by emotional exhaustion, depersonalization, and a diminished sense of efficacy, typically arising from chronic occupational stressors such as excessive workload and insufficient support [12, 13], but also from non-occupational stressors [14]. Burnout has been consistently linked to adverse outcomes for healthcare professionals (e.g., poor mental and physical health, and cognitive difficulties), organisations (e.g. increased cost, medical errors, and staff turnover) and patients (e.g., diminished care quality and satisfaction) [15–17].

Despite the robust literature on burnout and compassion fatigue as adverse outcomes, and compassion satisfaction as a positive one, prevalence remains problematic, challenging to measure and to improve. Levels among healthcare workers have been reported as problematic in various settings and countries, with burnout and compassion fatigue levels often at moderate to high, and compassion satisfaction usually at moderate levels [4, 18–20]. Direct comparison is, however, challenging due to varied conceptualisations and measurement approaches used for all three components [8, 16].

Efforts to improve Professional Quality of Life (ProQOL) among healthcare professionals have utilised a wide range of interventions, and their outcomes remain notably heterogeneous [4, 11, 15, 21, 22]. While individual-focused interventions—such as those targeting emotional intelligence or stress management skills—are more frequently implemented, growing evidence indicates that organisational or multi-component interventions tend to yield more substantial and sustained benefits for well-being and burnout reduction [23–27]. However, the structure and content of combined interventions vary considerably across settings, complicating the interpretation of their effectiveness and limiting the generalisability or transferability of best practices between organisations [15, 16, 22].

Whilst it is not expected for research to define all factors and interactions influencing positive outcomes in a specific context, exploring why and how some factors work in particular contexts can inform the design and development of context-sensitive interventions and improve their chances of success [28]. Unfortunately, there is limited empirical research investigating when and how active components of individual-level and organisational-level interventions interact to influence the distinct components of ProQOL [15, 17, 29, 30]. When such combinations are explored, there is a recurring risk of drawing misleading or unwarranted inferences about the nature and direction of variable interactions, particularly when proposed models are not solidly anchored in current empirical evidence and an integrated theoretical framework [31–33].

### A theoretical-empirically supported mediated moderation model

To address critical gaps in the Professional Quality of Life (ProQOL) literature, the present study explores the relationships between selected individual and organisational factors and ProQOL within an ecological multilevel framework. The framework is adapted from Bronfenbrenner’s Ecological Systems Theory [34], which explains that a series of interconnected environmental systems shape an individual’s development and that factors within and between systems interact dynamically over time. Within this overarching framework, individual factors (skills, abilities, behaviours) were placed at the core, and hypothesised effects and conditional context-dependent interactions shaping ProQOL were explored using empirical evidence and available theories that support the proposed direction of influence.

#### Mediation

Individual factors considered include emotional intelligence and self-care. Emotional intelligence is the ability to accurately perceive, understand, and regulate 1’s own and others’ emotions [35]. Emotional intelligence is a strong predictor of positive outcomes [36], and facilitates emotional processing, resilience and adaptive coping [37–39].

Self-care refers to the attitudes and practices that promote and maintain wellbeing and health, both at work and outside work (e.g., perceptions of the value of self-care, strategies such as personal habits or boundary setting at work) [40, 41]. Self-care is a strong individual predictor of positive outcomes include PROQOL [36, 42, 43]. Empirical evidence has suggested that self-care serves as a mediator transmitting the beneficial effects of emotional intelligence to ProQOL outcomes [44]. The authors use the theory of Salutogenesis [45, 46] to explain the role of self-regulation mechanisms (e.g., emotional intelligence) in activating and mobilising resources, such as self-care, to mitigate stressors and enhance well-being.

#### Moderation

Regarding organisational factors, empirical evidence has shown that emotional job demands may intensify the risk of burnout and compassion fatigue via emotional depletion, especially when resources are limited [29]. Emotional job demands is defined as the frequency and intensity of exposure to others’ suffering and distress when working [47]. According to the Compassion Fatigue Model [5], individuals’ coping and emotional regulation abilities are crucial when providing effective, sustained empathic engagement at work to mitigate the risk of emotional depletion- and consequently compassion fatigue and burnout. Therefore, the emotional demands of different types of work (TOW) will impact the positive effects of regulation abilities, shaped by emotional intelligence and self-care, on compassion fatigue and burnout. Work roles were categorised as direct providers, non-direct providers, and managers to reflect established distinctions in stress exposure and support dynamics [48, 49].

Another organisational factor, workplace social support, has also been shown to impact the positive effects of self-care on professional quality of life [50], to buffer the adverse effects of job stressors on life satisfaction [51], and to interfere with the enactment of emotional intelligence to improve work performance [38] and satisfaction [52]. Workplace Social Support is the emotional, informational, and practical help from supervisors, colleagues, and organisational community [51]. The Job Demands-Resources (JDR) Model [15] theorises that the imbalance between resources and demands at work can lead to strains. Therefore, workplace social support (resource) and type of work (emotional demands) are contextual factors that condition, buffer, or amplify the impact of individual resources, therefore supporting their moderating role on the direct or indirect (via self-care) impact of emotional intelligence on ProQOL.

#### Covariates

Due to their documented but inconsistent associations with emotional intelligence, self-care, and ProQOL across working contexts, gender and age were considered potential covariates in this study [44, 53–56]. 

### The present study

While individual strengths like emotional intelligence and engagement in self-care are protective, few studies clarify when and how these capacities interact with organisational variables—workplace social support and emotional job demands—to influence ProQOL’s three components. This study advances understanding of mediation and moderation mechanisms underlying these relationships.

**Fig. 1:**
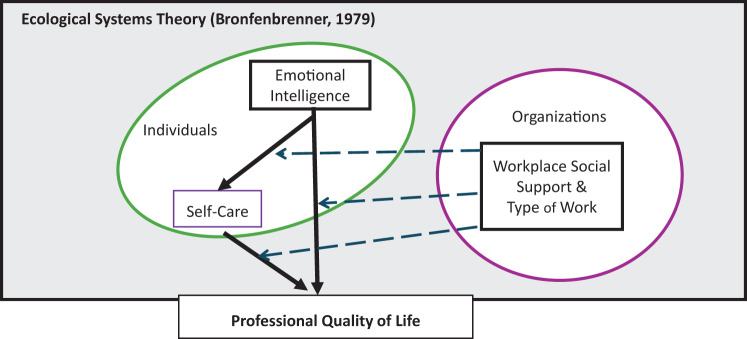
Moderated Mediation Model. Conceptual model illustrating how individual (emotional intelligence, self-care) and contextual (support, demands) factors influence each other, affecting all ProQOL components. The moderated mediation model is situated within Bronfenbrenner’s Ecological Theory (1979) to acknowledge that not all relevant factors and potential reciprocal interactions have been considered in this study. Arrows indicate the hypothesised directional effects of the proposed interactions: straight arrows represent direct or indirect (mediated) effects via self-care, while dashed arrows denote interaction effects (moderation)

Three hypotheses have been proposed: 1) There is a relationship between the variables; 2) Self-care mediates the relationship between emotional intelligence and professional quality of life; and 3) Workplace social support and type of work moderate the direct and indirect relationships. The study seeks to clarify how and under what conditions individual capacities combine with organisational factors to sustain compassion satisfaction and reduce burnout and compassion fatigue using a moderated mediation model of interactions Fig. 1.

## Methods

### Aim, design, and setting

This cross-sectional correlational study explored hypothesised relationships among multiple variables related to Professional Quality of Life (ProQOL) within a convenience sample of healthcare workers. An online survey was employed, which enabled efficient collection of new data from a representative sample at a specific point in time and supported examination of complex inter-variable relationships [57–59]. The study was conducted within the Central Coast Local Health District (CCLHD), a large public health authority providing both hospital-based and community care services across the Central Coast region of Australia, comprising over 5,500 full-time equivalent (FTE) staff.

### Participants

A total of 343 healthcare workers (mean age = 46.02, SD = 11.48) participated in the study. The inclusion criteria were (1) being over 18 years old and (2) currently working at the Central Coast Local Health District (CCLHD), a public health authority delivering hospital and community-based services across the Central Coast region of Australia. Prior to analysis, a post hoc power calculation confirmed that the sample size (*n* = 343) exceeded the minimum requirement for detecting a medium effect (f^2^ = 0.15) in multiple regression (19 predictors, α = 0.05, power = 95%), as calculated by G*Power 3.1, which indicated a minimum sample of 217.

Among the participants, 275 (80.2%) identified as females, 56 (16.3%) as males, 182 (53.1%) provided direct clinical services, 68 (19.8%) provided non-direct clinical services, and 72 (21.0%) were managers of direct providers. The study included healthcare workers from a single Local Health District in New South Wales (NSW), Australia. The NSW health workforce—about 205,000 nurses, allied health professionals, and medical practitioners—is predominantly female (average age ~43 years) and spans diverse clinical and managerial roles [60]. Participants worked in a system guided by the CORE values of Collaboration, Openness, Respect, and Empowerment, which promote psychological safety, inclusive leadership, and staff wellbeing [60]. Role differences—direct providers, non-direct providers, and managers—entailed varying job demands and social support. This demographic and organisational context is integral to interpreting professional quality of life findings.

### Material and apparatus

A battery of validated measurement instruments was distributed through a secure online survey platform (JISC surveys). All instruments demonstrated acceptable reliability (Cronbach’s alpha above 0.70) in either source studies or the present sample.

ProQOL was assessed using the Short Professional Quality of Life Compassion Satisfaction Scale (sProQOL) [61] in which individuals indicate their agreement with 9 statements using a 5-point Likert scale (1 = never to 5 = very often). From this, burnout (BO), compassion fatigue (CF) and compassion satisfaction (CS) scores are derived. Cronbach values were 0.810 (BO), 0.763 (CF), and 0.737 (CS) [61].

Emotional intelligence was assessed using the Wong and Law Emotional Intelligence Scale [62] in which individuals indicate their agreement with 16 statements on a 7-point Likert scale (1 = strongly disagree to 7 = strongly agree). From this, global scores, and four-factor scores, for self-emotional appraisal (SEA), others’ emotional appraisal (OEA), use of emotions (UOE) and regulation of emotions (ROE), are derived. Cronbach values were 0.87 (SEA), 0.90 (OEA), 0.84 (UOE), and 0.83 (ROE) [62].

The self-care practice was assessed using 13 out of 18 items from the Self-Care Practice Scale (SCa) [63] and 5 items adapted from the Mindful Self-Care Scale–Brief (MSCS) questionnaire [64]. Respondents endorsed how often they engaged in specific behaviours in 18 self-care practices using a five-point Likert scale (1 = never to 5 = very often). From this, self-care practice (SCpra), self-care personal practice (SCpras) and self-care professional practice (SCpraf) scores are derived. Cronbach’s alphas were 0.93 (SCpra), 0.89 (SCpras), and 0.87 (SCpraf) [65]. Cronbach’s coefficient alphas for the MSCS scale was 0.89 [64].

Self-care perceptions were assessed using the Self-care Perceptions Scale (SCc) [66]. In this scale, respondents indicated their agreement to 11 statements stating their appreciation, value, and/or awareness of self-care using a 5-point Likert scale (0 = strongly disagree to 5 = strongly agree), with 4 items reversed scored. From this, self-care perception (SCperc), self-care perception personal (SCpercs) and self-care perception professional (SCpercf) scores are derived. Cronbach value was 0.76 (SCperc) [66]. A global score for self-care (SC) was derived from the addition of the SCc and SCpra scores Table 1.

**Table 1:** Samples of all instruments used in this study

Construct and tool	Subscales	Example item
Professional Quality of Life: ProQOL (9 items)	Compassion fatigue	“I feel depressed as a result of my work as a helper”.
	Compassion satisfaction	“My work makes me feel satisfied”
	Burnout	“I feel overwhelmed by the amount of work, or the size of the work load I have to deal with”
Emotional intelligence: WLEIS (16 items)	Self-emotions appraisal	“I have a good sense of why I have certain feelings most of the time”
	Other emotions appraisal	“I have good understanding of the emotions of people around me”
	Use of emotions	“I always set goals for myself and then try my best to achieve them”
	Regulation of emotions	“I am quite capable of controlling my own emotions”
Self-Care Perception: SCc (11 items)	Personal	“I value self-care”
	Professional	“My current employer effectively teaches me how to engage in self-care”
Self-Care Practice 1: 13 items from SCPS	Personal	“I spend quality time with people I care about”
	Professional	“I attend to feelings of being overwhelmed with my work”
Self-Care Practice 2: 5 items from MSCS	Personal	“I engage in supportive and comforting self-talk”
	Professional	“I experienced meaning and/or a larger purpose in my work life (e.g., for a cause).”
Workplace Social Support: 6 items from COPOQ III	Supervisor support	“How often is your immediate superior willing to listen to your problems at work. if needed?”
	Colleague support	“How often do you get help and support from your colleagues, if needed?”
	Sense of community	“Is there a good atmosphere between you and your colleagues?”

Examples of items reproduced from the Short Professional Quality of Life Compassion Satisfaction Scale (sProQOL) [61], the Wong and Law Emotional Intelligence Scale [62], Self-Care Practice Scale (SCa) [63], Self-care Perceptions Scale (SCc) [66], and Third Version of the Copenhagen Psychosocial Questionnaire COPSOQ III [67]

Workplace social support was assessed using selected items from the domain ‘interpersonal relations and leadership’ from the Third Version of the Copenhagen Psychosocial Questionnaire COPSOQ III [67]. Individuals indicated the frequency of 6 types of experiences in the last 30 days using a 5-point Likert type scale (1 = Never/hardly ever to 5 = strongly always). From this, global scores for workplace social support are derived from 3-factor scores: social support from colleagues (WScol), social support from supervisor (Wssup), and a sense of community at work (WScom). Cronbach’s alpha was 0.87 (WScol), 0.81 (WSsup), and 0.79 (WScom) [67].

Socio-demographic information was collected using three questions to identify participants’ age, gender, and type of work (provider of direct or indirect care, or manager). Samples of all instruments used in this study can be found in Table 1.

### Procedure

The Central Coast Local Health District (CCLHD) Research Officer facilitated initial contact by introducing the principal researcher to various department and unit heads at Gosford and Wyong Hospitals. The researcher provided a standardized briefing to directors of key divisions about the study’s aims, methodology, and logistics, seeking their support for dissemination and staff engagement. Formal approval for study dissemination was obtained from eleven directors representing key CCLHD divisions, including the Nurse and Midwifery Directorate, Medical Services, Community Wellbeing and Allied Health, Mental Health, Population Health Improvement, Acute Care Services, Palliative Care, Workforce and Culture, and the Public Health Unit.

On October 30th, 2023, these departments distributed emails containing an electronic study poster to all eligible staff. A reminder email was sent on November 11th, 2023. Additionally, the study was publicized through physical posters placed in staff-only common areas and via internal communication channels such as the CCLHD general broadcast (November 3rd, 2023), Wyong Hospital’s ‘Grapevine’ magazine (November 3rd, 2023), and Gosford Hospital’s ‘The Pulse’ magazine (November 17th, 2023). The study poster included a link and QR code directing participants to the Participant Information Sheet, Consent Form, and the online survey hosted securely on the Brunel University JISC platform. Participation was voluntary, anonymous, and confidential, with no monetary or material compensation offered.

The survey remained open for three weeks, from October 30th to November 20th, 2023. Participants could complete the survey at their convenience during this timeframe. The estimated completion time was between 10 and 20 minutes. Prior to full deployment, the survey was pilot tested with two co-authors and supervisors (ZT and WA), primarily to refine language and ensure cultural and contextual appropriateness for the Australian healthcare setting.

To ensure data integrity, only one response per individual was permitted, with duplicate entries prevented by JISC IP-based/cookie-based duplicate protection. Participants were informed of their right to withdraw at any point before final survey submission in the participant Information Sheet and before signing the consent form in the JISC platform. Data confidentiality and security were maintained according to institutional policies. All data were securely stored on password-protected Brunel University servers accessible only to the research team. Responses were anonymised upon collection, with no personal identifiers recorded.

### Data analysis

Survey data were exported to IBM SPSS (Mac OS, Version 29.0.0) for statistical analysis. Significance was set at *p* < 0.05. Preliminary data exploration, sample characteristics and relationships between variables were investigated using descriptive statistics, bivariate correlations, and group comparisons. As items were added or taken from already validated questionnaires, the reliability of the scales used for this study was calculated using Cronbach’s Alpha.

Primary analyses comprised mediation and moderated mediation using the PROCESS macro [68]^1^, with model 4 (for mediation) and model 76 (for moderated mediation). The significance of the indirect effects was tested using a bias-corrected bootstrapping confidence interval. Gender and age were adjusted for in all inferential analyses. Subscale relationships were explored where possible.

## Results

### Data screening and descriptive statistics

The dataset was assessed for missing values, outliers, and normality assumptions. The size, type, and pattern of missing values were deemed acceptable. Outliers were examined and confirmed as valid responses. The full dataset was retained with pairwise and listwise deletion applied selectively for specific analyses. As the normality assumption was not met, appropriate non-parametric tests were employed.

Gender distribution by age and type of work is presented in Table 2, and descriptive statistics for core variables are in Table 3. Scale item mean scores are reported in Supplement B. No out-of-range values were observed, and standard deviations were consistently below 2. Reliability analyses showed adequate internal consistency; Cronbach’s alpha was generally above 0.7, and McDonald’s Omega (recommended for non-parametric data [69, 70], confirmed reliability (see Table 3).

**Table 2:** Gender distribution according to participants’ age and type of work

Category		Gender ^a^		Female		Male	
Age (years)							
	Mean	46.0		46.0		46.4	
	Standard deviation	11.5		11.2		12.9	
	Minimum	21		21		25	
	Maximum	90		71		90	
	Total number of elements	324		268		56	
Type of work		n	%	n	%	n	%
	Direct providers	175	56	145	46	30	10
	Managers	71	23	64	20	7	2
	Non-direct providers	67	21	51	16	16	5
	Total number of elements	313	100	260	83	53	17

*N* = 331 for age and *N* = 321 for type of work. The total number of elements in a sample (N) reflects the number of participants answering a given question. It differs between categories because the missing data was managed using listwise deletion. The elements in each subgroup (n) of the full sample reflect the number of participants who selected the same response option (e.g. woman). aGender includes participants who selected “woman” or “man” options only to protect the anonymity of the very few participants who selected other options

**Table 3:** Means, standard deviations, and reliability measures scores

Scales^a^	Scores^b^	M	SD	Items	α	ω
Professional Quality of Life						
Burnout	9.86	3.29	0.94	3	0.80	0.80
Compassion fatigue	7.32	2.44	0.89	3	0.78	0.79
Compassion satisfaction	11.54	3.85	0.77	3	0.83	0.83
Emotional intelligence	89.89	5.61	0.71	16	0.88	0.88
Others’ emotion appraisal	23.09	5.77	0.81	4	0.81	0.82
Regulation of emotion	21.70	5.43	1.13	4	0.90	0.90
Self-emotion appraisal	23.09	5.77	0.89	4	0.86	0.86
Use of emotion	21.94	5.48	1.02	4	0.80	0.80
Self-care	NA	3.26	0.49	29	0.89	0.88
Self-care perception	NA	3.08	0.55	11	0.76	0.72
Self-care perception personal	NA	3.42	0.61	5	0.62	0.61
Self-care perception professional	NA	2.79	0.71	5	0.79	0.77
Self-care practice	60.31	3.35	0.54	18	0.87	0.87
Self-care practice personal	30.32	3.37	0.68	9	0.87	0.87
Self-care practice professional	29.96	3.33	0.56	9	0.77	0.76
Workplace social support	NA	3.64	0.83	6	0.85	0.83
Work sense of community	NA	3.80	0.93	2	0.80	NA
Work colleague support	NA	3.79	0.91	2	0.83	NA
Work Supervisor support	NA	3.33	1.20	2	0.87	NA

*N* = 343. Reliability measures tested included Cronbach’s Alpha (α) and McDonald’s Omega (ω), which were not applicable (NA) on scales with fewer than three items. a Measures used a 5-point Likert-type scale except for emotional intelligence which had a 7-point Likert-type scale. b The scores of professional quality of life components were considered high for burnout and compassion fatigue and moderate for compassion satisfaction using published guidelines [61]

### Hypothesis 1 − relationships between variables

Spearman’s correlation and group comparison analyses supported Hypothesis 1, confirming significant relationships among emotional intelligence, self-care, workplace social support, type of work, and all three ProQOL components.

As shown in Table 4, all six scale variables correlated significantly (*p* < 0.001). Predictors were positively interrelated and positively correlated with compassion satisfaction. Burnout and compassion fatigue were strongly positively correlated (rho = 0.730), and both negatively correlated with compassion satisfaction and the predictor variables.

**Table 4:** Bivariate Spearman’s rho correlation coefficients for scales and subscales

	Variables	1	2	3	4	5	6	7	8	9	10	11	12	13	14	15	16	17	18	19
1	Age in years																			
2	Burnout	−0.014																		
3	Compassion Fatigue	0.047	0.730^**^																	
4	Compassion Satisfaction	0.013	−0.497^**^	−0.377^**^																
5	Emotional Intelligence	0.099	−0.231^**^	−0.273^**^	0.294^**^															
6	Self-Care Global	−0.043	−0.489^**^	−0.418^**^	0.488^**^	0.474^**^														
7	Workplace Social Support	−0.073	−0.424^**^	−0.381^**^	0.380^**^	0.246^**^	0.472^**^													
8	Others’ emotion appraisal	0.031	0.029	−0.02	0.110^*^	0.573^**^	0.148^**^	0.122^*^												
9	Regulation of emotion	0.046	−0.232^**^	−0.233^**^	0.245^**^	0.760^**^	0.359^**^	0.195^**^	0.242^**^											
10	Self-emotion appraisal	0.065	−0.214^**^	−0.259^**^	0.215^**^	0.673^**^	0.427^**^	0.212^**^	0.333^**^	0.398^**^										
11	Use of emotion	0.089	−0.182^**^	−0.224^**^	0.276^**^	0.704^**^	0.404^**^	0.216^**^	0.225^**^	0.385^**^	0.339^**^									
12	Self-Care Perceptions	−0.039	−0.505^**^	−0.434^**^	0.432^**^	0.311^**^	0.785^**^	0.461^**^	0.093	0.266^**^	0.323^**^	0.213^**^								
13	Self-Care Perception Personal	−0.028	−0.303^**^	−0.264^**^	0.237^**^	0.206^**^	0.611^**^	0.235^**^	0.031	0.201^**^	0.254^**^	0.127^*^	0.727^**^							
14	Self-Care Perception Professional	−0.037	−0.500^**^	−0.431^**^	0.437^**^	0.285^**^	0.669^**^	0.483^**^	0.111^*^	0.218^**^	0.269^**^	0.205^**^	0.874^**^	0.327^**^						
15	Self-Care Practice	−0.037	−0.394^**^	−0.330^**^	0.433^**^	0.485^**^	0.945^**^	0.404^**^	0.149^**^	0.353^**^	0.406^**^	0.444^**^	0.560^**^	0.461^**^	0.468^**^					
16	Self-Care Personal Practice	−0.039	−0.302^**^	−0.239^**^	0.311^**^	0.454^**^	0.824^**^	0.285^**^	0.103	0.366^**^	0.376^**^	0.427^**^	0.468^**^	0.497^**^	0.324^**^	0.881^**^				
17	Self-Care Professional Practice	−0.035	−0.389^**^	−0.338^**^	0.478^**^	0.373^**^	0.801^**^	0.430^**^	0.154^**^	0.227^**^	0.325^**^	0.331^**^	0.496^**^	0.290^**^	0.490^**^	0.830^**^	0.499^**^			
18	Work Sense of Community	0.031	−0.377^**^	−0.315^**^	0.403^**^	0.238^**^	0.374^**^	0.802^**^	0.126^*^	0.213^**^	0.184^**^	0.202^**^	0.337^**^	0.191^**^	0.343^**^	0.342^**^	0.242^**^	0.365^**^		
19	Work Colleague Social Support	−0.071	−0.244^**^	−0.228^**^	0.237^**^	0.139^**^	0.331^**^	0.801^**^	0.064	0.092	0.136^*^	0.151^**^	0.258^**^	0.118^*^	0.278^**^	0.315^**^	0.213^**^	0.360^**^	0.642^**^	
20	Work Supervisor Social Support	−0.113^*^	−0.375^**^	−0.349^**^	0.275^**^	0.225^**^	0.420^**^	0.815^**^	0.088	0.175^**^	0.199^**^	0.207^**^	0.468^**^	0.237^**^	0.491^**^	0.327^**^	0.252^**^	0.320^**^	0.431^**^	0.442^**^

*. Correlation is significant at the 0.05 level (2-tailed), **. Correlation is significant at the 0.01 level (2-tailed). Age N = 331, other variables N = 343

The strongest correlations involved burnout’s associations: positively with compassion fatigue (rho = 0.730, *p* < 0.001) and negatively with compassion satisfaction (rho = −0.497, *p* < 0.001), self-care (rho = −0.489, *p* < 0.001), and workplace social support (rho = −0.424, *p* < 0.001). Self-care showed moderate negative correlation with compassion fatigue (rho = −0.418, *p* < 0.001) and positive correlations with compassion satisfaction (rho = 0.488, *p* < 0.001), EI (rho = 0.474, *p* < 0.001), and workplace social support (rho = 0.472, *p* < 0.001). Compassion fatigue and compassion satisfaction had a moderate negative relationship (rho = −0.377, *p* < 0.001), with similar associations to workplace social support (negative and positive, respectively; approx. rho = ±0.38).

Subscale-level correlations followed similar patterns, except for some specific components such as emotion regulation and appraisal. The covariate age was not significantly correlated with the core scales except for a weak positive correlation with supervisor social support (rho = 0.113, *p* = 0.005).

#### Group comparisons

Kruskal-Wallis tests examined differences across types of work (direct providers, managers, non-direct providers). Significant differences were found on three of six scales (Table 5), supporting Hypothesis 1. Direct providers reported higher compassion fatigue and lower workplace social support (particularly in sense of community and supervisor support) compared to other groups (χ^2^ (2) = 7.554, *p* = 0.02; χ^2^ (2) = 12.096, *p* = 0.02). Managers scored significantly higher on emotional intelligence, especially in self-emotion appraisal and use of emotions (χ^2^ (2) = 8.948, *p* = 0.011).

**Table 5:** Significant differences in scores between type of work groups

Variables	Type of work	n	Mean Rank	df	*χ*^*2*^	p
Compassion fatigue	Direct providers	182	173.90	2	7.55	0.002^**^
	Managers	72	146.42			
	Non-direct providers	68	144.29			
Emotional intelligence	Direct providers	182	151.63	2	8.95	0.01^**^
	Managers	72	190.06			
	Non-direct providers	68	157.67			
- Self-emotions appraisal	Direct providers	182	153.50	2	6.82	0.033^*^
	Managers	72	186.33			
	Non-direct providers	68	156.62			
- Use of emotions	Direct providers	182	150.89	2	10.84	0.004^**^
	Managers	72	192.90			
	Non direct providers	68	156.65			
Workplace social support	Direct providers	182	145.71	2	12.09	0.02^**^
	Managers	72	181.98			
	Non-direct providers	68	182.08			
- Sense of community	Direct providers	182	148.34	2	8.72	0.013^*^
	Managers	72	177.58			
	Non-direct providers	68	179.70			
- Supervisor support	Direct providers	182	144.61	2	14.24	< 0.001^***^
	Managers	72	186.65			
	Non-direct providers	68	180.08			

*N* = 323. A Kruskal-Wallis test was performed to compare differences in the scales’ mean rank scores between types of work groups. Only significant results have been reported in this table. Complete test results are available upon request. * *p* < 0.05. **p < 0.01.***p < 0.001

No significant association was found between gender and work group (χ^2^ (2, *N* = 313) = 4.831, *p* = 0.089). Mann-Whitney U tests revealed no gender differences across most variables except for emotional intelligence (see Table 6).

**Table 6:** Significant differences in scores between females and males

Variable	Mean Rank		U	p	z	*r*^*a*^
	Woman	Man				
Emotional intelligence	172.91	132.06	5799.50	0.004^**^	−2.91	−0.16
- Self-emotion appraisal	172.80	132.61	5830.00	0.004^**^	−2.95	−0.16
- Other’s emotion appraisal	177.06	111.70	4659.00	< 0.001^***^	−4.71	−0.26
Self-care perception	170.57	143.57	6444.00	0.054	−1.93	−0.11
- Self-care perception professional	170.99	141.47	6326.50	0.035^*^	−2.11	−0.12

*N* = 331. A Mann-Whitney U test was performed to compare differences in the scales and subscales’ mean rank scores between females and males. Only significant results have been reported in this table. Complete test results are available upon request. aCalculated using *r* = Z/√N. * *p* < 0.05. **p < 0.01. ***p < 0.001

### Hypothesis 2 – mediation analysis

Mediation analyses supported Hypothesis 2, indicating that self-care significantly mediated the relationship between EI and ProQOL components.

As shown in Table 7, emotional intelligence was a significant positive predictor of self-care (*p* < 0.001). Regarding subscales (Table C.1 Supplement C), the direct effects of self-care professional perception and practice effects were comparably higher than self-care personal perception and practice on burnout (respectively,-.609 and −0.588) vs. (−0.419 and −0.314), all *p* < 0.000).; on compassion fatigue (respectively,-.459 and −0.452, both *p* < 0.000) vs. (−0.294, *p* < 0.000 and −0.157), *p* < 0.01).; and on compassion satisfaction (respectively,(−0.445 and −0.626) vs. (−0.227 and −0.285), all *p* < 0.000).

**Table 7:** Results of mediation analysis for path A

DV: Self-care (*mediator variable model) R*^2^ = 0.205, *p* < 0.001, df(3;320)					
		B	SE	t	p
Constant		1.623	0.238	6.84	0.000
Emotional intelligence (IV) (a path)		0.316***	0.035	8.94	0.000
Controlled variables:	Gender	0.003	0.065	0.043	0.966
	Age	−0.003	0.002	−1.44	0.150

N = 324. p*** < 0.001, CI*

**Fig. 2:**
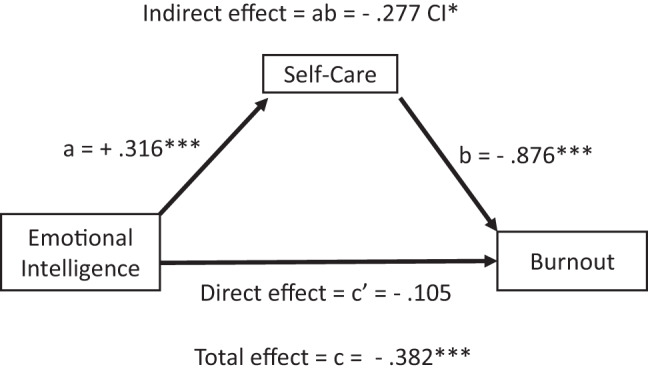
Effects mediation analysis for burnout. Fig. 2 shows that the total and indirect effects (mediated via self-care) of emotional intelligence on burnout and the effects of self-care on burnout were significant. The direct effect of emotional intelligence on burnout was not significant. Arrows indicate directional effects: straight arrows represent direct or indirect (mediated) effects via self-care. Path a represents the direct effects of emotional intelligence on self-care, path b the direct effects of self-care on burnout and path c’ the direct effects of emotional intelligence on burnout. Path ab represents the indirect effects of emotional intelligence via self-care on burnout (path a*b), and path c the total effects of emotional intelligence on burnout (path ab + c’). *N* = 324, * *p* < 0.05. **p < 0.01.***p < 0.001. p*** < 0.001, CI* does not overlap with zero

**Fig. 3:**
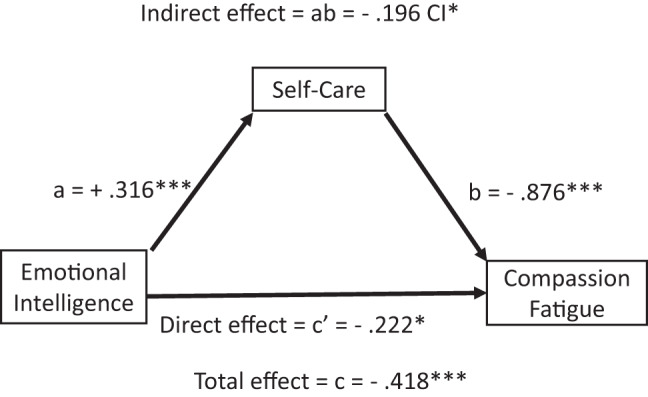
Effects mediation analysis for compassion fatigue. The figure shows that the total, direct, and indirect effects (mediated via self-care) of emotional intelligence on compassion fatigue and the effects of self-care on compassion fatigue were significant. Arrows indicate directional effects: straight arrows represent direct or indirect (mediated) effects via self-care. Path a represents the direct effects of emotional intelligence on self-care, path b the direct effects of self-care on compassion fatigue and path c’ the direct effects of emotional intelligence on compassion fatigue. Path ab represents the indirect effects of emotional intelligence via self-care on compassion fatigue (path a*b), and path c the total effects of emotional intelligence on compassion fatigue (path ab + c’). N = 324, p*** < 0.001, p* < 0.005, CI* does not overlap with zero

**Fig. 4:**
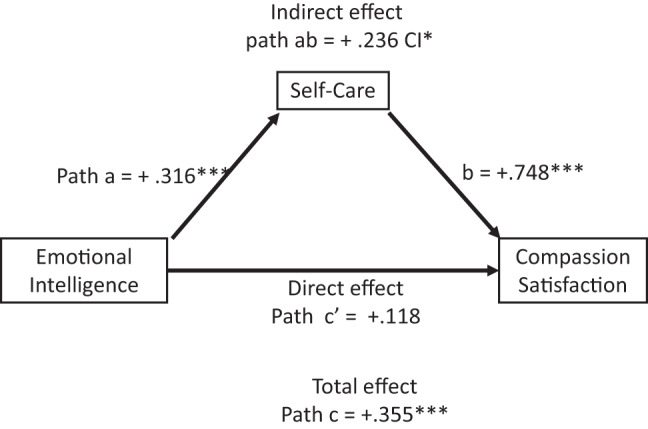
Effects mediation analysis for compassion satisfaction. The figure shows that the total and indirect effects (mediated via self-care) of emotional intelligence on compassion satisfaction and the effects of self-care on compassion satisfaction were significant. The direct effects of emotional intelligence on compassion satisfaction were not significant. Arrows indicate directional effects: straight arrows represent direct or indirect (mediated) effects via self-care. Path a represents the direct effects of emotional intelligence on self-care, path b the direct effects of self-care on compassion satisfaction and path c’ the direct effects of emotional intelligence on compassion satisfaction. Path ab represents the indirect effects of emotional intelligence via self-care on compassion satisfaction (path a*b), and path c the total effects of emotional intelligence on compassion satisfaction(path ab + c’). N = 324, * p < 0.05. **p < 0.01.***p < 0.001. p*** < 0.001, CI* does not overlap with zero

The total and indirect effects of emotional intelligence on burnout (Table 8 and Fig. 2), compassion fatigue (Table 9 and Fig. 3), and compassion satisfaction (Table 10 and Fig. 4) were significant, with negative effects on burnout and compassion fatigue, and positive effects on compassion satisfaction. The direct effects of emotional intelligence were significant only for compassion fatigue. Regarding the subcomponents of emotional intelligence direct effects (Table C.1 Supplement C), it was notable that all but “other-emotional appraisal” were significant on compassion fatigue, whilst only “use of emotions” was significant on burnout and “regulating other emotions” for compassion satisfaction. Covariates gender and age were non-significant across all models.

**Table 8:** Results of mediation analysis for burnout

DV: Burnout (Dependent variable model) *R*^2^ = 0.499, *p* < 0.001, df(4;319)					
		B	SE	t	p
Constant		7.029	0.471	14.91	0.000
Emotional intelligence (Direct effect, c’ path)		−0.105	0.073	−1.43	0.153
Self-care (b path)		−0.876***	0.104	−1.52	0.000
Controlled variables:	Gender	1.185	0.121	−1.52	0.128
	Age	−0.001	0.004	−0.34	0.734
DV: Burnout (Total effect model) *R*^2^ = 0.285, *p* < 0.001, df(3;320)					
Constant		5.606	0.486	11.53	0.000
Emotional intelligence (Total effect)		−0.382***	0.072	−5.28	0.000
Controlled variables	Gender	−0.187***	0.134	−1.39	0.000
	Age	0.001	0.004	0.31	0.758
Mediation (through self-care)					
				Boostrapped IC	
		ab	BootSE	LLCI	LLCI
Indirect effect(s) of X on Y (ab path)		−0.277 CI*	0.060	−0.408	−0.177

N = 324, p*** < 0.001, CI* does not overlap with zero, level of confidence (IC) 95.0000, number of bootstrap samples 5000. * p < 0.05. **p < 0.01.***p < 0.001

**Table 9:** Results of mediation analysis for compassion fatigue

Dependent varriable model: Compassion fatigue *R*^2^ = 0.200, *p* = < 0.001, df(4;319)					
		B	SE	t	p
Constant		5.604	0.464	12.81	0.000
Emotional intelligence (Direct effect, c’ path)		−0.222**	0.072	−3.08	0.002
Self-care (b path)		−0.876***	0.104	−1.52	0.000
Controlled variables	Gender	−0.064	0.119	−0.540	0.586
	Age	0.004	0.004	1.019	0.309
DV: Compassion fatigue (Total effect model) *R*^2^ = 0.328, *p* < 0.001, df(3;320)					
		B	SE	*t*	*p*
Constant		4.599	0.457	10.07	0.000
Emotional intelligence (Total effect)		−0.418***	0.068	−6.15	0.000
Controlled variables:	Gender	−0.066	0.126	−0.526	0.599
	Age	0.006	0.004	1.43	0.153
Mediation (through self-care)					
				Boostrapped IC	
		ab	BootSE	LLCI	LLCI
Indirect effect(s) of X on Y (ab path)		−0.196 CI*	0.049	−0.307	−0.117

N = 324, p*** < 0.001, CI* does not overlap with zero, level of confidence (IC) 95.0000, number of bootstrap samples 5000. * p < 0.05. **p < 0.01.***p < 0.001

**Table 10:** Results of mediation analysis for compassion satisfaction

Dependent variable model (*R*^2^ = 0.276, *p* < 0.001, df(4; 319))					
		B	SE	t	p
Constant		0.616	0.387	1.59	0.113
Emotional intelligence (Direct effect, c’ path)		0.118	0.060	1.964	0.050
Self-care (b path)		0.748***	0.085	8.78	0.000
Controlled variables	Gender	0.010	0.100	0.101	0.919
	Age	0.002	0.003	0.756	0.450
Total effect model - *R*^2^ = 0.101, *p* < 0.001, df(3;320)					
		B	SE	*t*	*p*
Constant		1.899	0.402	4.54	0.000
Emotional intelligence (Total effect)		0.355***	0.060	5.92	0.000
Controlled variables:	Gender	0.012	0.111	0.110	0.912
	Age	0.000	0.004	0.045	0.964
Mediation (through self-care)					
				Boostrapped IC	
		ab	BootSE	LLCI	LLCI
Indirect effect(s) of X on Y (ab path)		0.236 CI*	0.043	0.163	0.276

N = 324, p*** < 0.001, CI* does not overlap with zero, level of confidence (IC) 95.0000, number of bootstrap samples 5000. * p < 0.05. **p < 0.01.***p < 0.001

### Hypothesis 3 − moderated mediation analysis

Moderation analyses supported Hypothesis 3, confirming that workplace social support and type of work moderated both the direct and indirect effects of EI on ProQOL. Gender and age did not significantly influence any interactions.

#### Conditional indirect effects of EI on ProQOL via self-care

The conditional indirect effects of EI on ProQOL components via self-care were significant (Table 11). Significant conditional indirect effects, particularly for workplace social support, were demonstrated by probing at moderator levels low (−1 SD), moderate (mean), and high (+1 SD) (Table 12), following recommended moderation analysis practices [71]. Prior studies confirm these cut-points capture meaningful variation in social support affecting healthcare workers’ quality of life [51, 72].

**Table 11:** Conditional indirect effects of EI on outcomes

Conditional Effects on DV	Burnout				Compassion Fatigue				Compassion Satisfaction			
	B	SE	t	p	B	SE	t	p	B	SE	t	p
Emotional intelligence (EI)	−0.151	0.111	−1.355	0.177	−0.203	0.113	−1.797	0.073	0.228*	0.095	2.410	0.017
Self-care (SC)	−0.579***	0.141	−4.097	0.000	−0.357*	0.143	−2.488	0.013	0.530***	0.120	4.417	0.000
Workplace social support (WPSS)	−0.357***	0.064	−5.564	0.000	−0.272***	0.065	−4.170	0.000	0.257***	0.054	4.716	0.000
Interaction EI*WPSS	0.039	0.066	0.592	0.555	0.031	0.067	0.461	0.645	0.080	0.056	1.431	0.154
Interaction SC*WPSS	−0.086	0.109	−0.788	0.431	0.078	0.111	0.700	0.484	−0.024	0.093	−0.263	0.793
Z1 (Type of work dummy 1)	0.005	0.118	0.04	0.963	0.003	0.120	0.024	0.981	−0.186	0.100	−1.863	0.064
Z2 (Type of work dummy 2)	0.112	0.115	0.97	0.334	−0.126	0.117	−1.073	0.284	−0.303**	0.098	−3.095	0.002
Interaction EI*Z1	0.130	0.201	0.47	0.518	−0.077	0.204	−0.377	0.706	−0.130	0.170	−0.765	0.445
Interaction EI*Z2	0.485*	0.199	2.443	0.015	0.275	0.201	1.368	0.172	−0.357*	0.168	−2.121	0.035
Interaction SC*Z1	−0.117	0.244	−0.481	0.631	−0.107	0.247	−0.434	0.665	−0.016	0.207	−0.078	0.938
Int SC*Z2	−0.419	0.300	−1.400	0.162	−0.527	0.304	−1.735	0.084	0.124	0.254	0.488	0.626
Gender	−0.181	0.119	−1.519	0.130	−0.077	0.121	−0.636	0.526	0.061	0.101	0.605	0.546
Age	−0.003	0.004	−0.883	0.378	0.002	0.004	0.607	0.544	0.006	0.003	1.705	0.089
Unconditional interaction	R2-chng	F	df	p	R2-chng	F	df	p	R2-chng	F	df	p
Interaction EI*WPSS	0.001	0.350	(1;292)	0.555	0.001	0.212	(1;292)	0.645	0.005	2.048	(1;292)	0.154
Interaction EI*TOW	0.013	3.002	(2;292)	0.051	0.006	1.328	(2;292)	0.266	0.010	2.251	(2;292)	0.107
Interaction BOTH (EI)	0.019*	2.847	(3,292)	0.038	0.008	1.092	(3,292)	0.354	0.011	1.681	(3,292)	0.171
Interaction SC*WPSS	0.001	0.622	(1;292)	0.431	0.001	0.491	(1;292)	0.484	0.000	0.069	(1;292)	0.793
Interaction SC*TOW	0.004	0.984	(2;292)	0.375	0.007	1.507	(2;292)	0.223	0.001	0.145	(2;292)	0.865
Interaction BOTH (SC)	0.007	1.078	(3,292)	0.359	0.008	1.058	(3,292)	0.367	0.001	0.114	(3,292)	0.952

The Table shows the conditional indirect effects of EI on outcomes. Note that the conditional direct effects of the type of work were reported as non-significant. Still, workplace social support significantly increased the effects, positively for compassion satisfaction (β = 0.257, *p* < 0.001), negatively for burnout (β = −0.357, *p* < 0.001), and compassion fatigue (β = −0.272, *p* < 0.001). Probing the interaction at the values of the moderators demonstrated that the conditional effects were not significant in all situations, except that the type of work modulated the effect of social support on compassion satisfaction for non-direct providers (β = −0.303, *p* = 0.02). * *p* < 0.05. **p < 0.01.***p < 0.001. *n* = 306, p*** < 0.001, p** < 0.01, p* < 0.005, CI* do not overlap zero

**Table 12:** Conditional indirect effects on outcomes at values of the moderator

Conditional Indirect Effects on Outcomes at Values of the Moderators				
Workplace social support	Type of work	Burnout	Compassion fatigue	Compassion satisfaction
−0.830	Direct providers	−0.101^*^	−0.084^*^	0.109^*^
	Managers	−0.095	−0.080	0.081
	Non-direct providers	−0.155	−0.158	0.113
0.000	Direct providers	−0.166^*^	−0.102^*^	0.152^*^
	Managers	−0.167^*^	−0.111^*^	0.123^*^
	Non-direct providers	−0.255^*^	−0.225^*^	0.167^*^
0.830	Direct providers	−0.244^*^	−0.109	0.19^*^
	Managers	−0.251^*^	−0.131^*^	0.161^*^
	Non-direct providers	−0.367^*^	−0.281^*^	0.217^*^

*n* = 306. The Table 12 shows the conditional indirect effects at values of workplace social support and type of work. Note that all providers reported positive increased effects on compassion satisfaction, and negative increased effects on burnout and compassion fatigue, with increased workplace social support. Level of confidence (CI) 95.0000, number of bootstrap samples 5000. * CI does not overlap with zero

##### Conditional effects of emotional intelligence on self-care

**Fig. 5:**
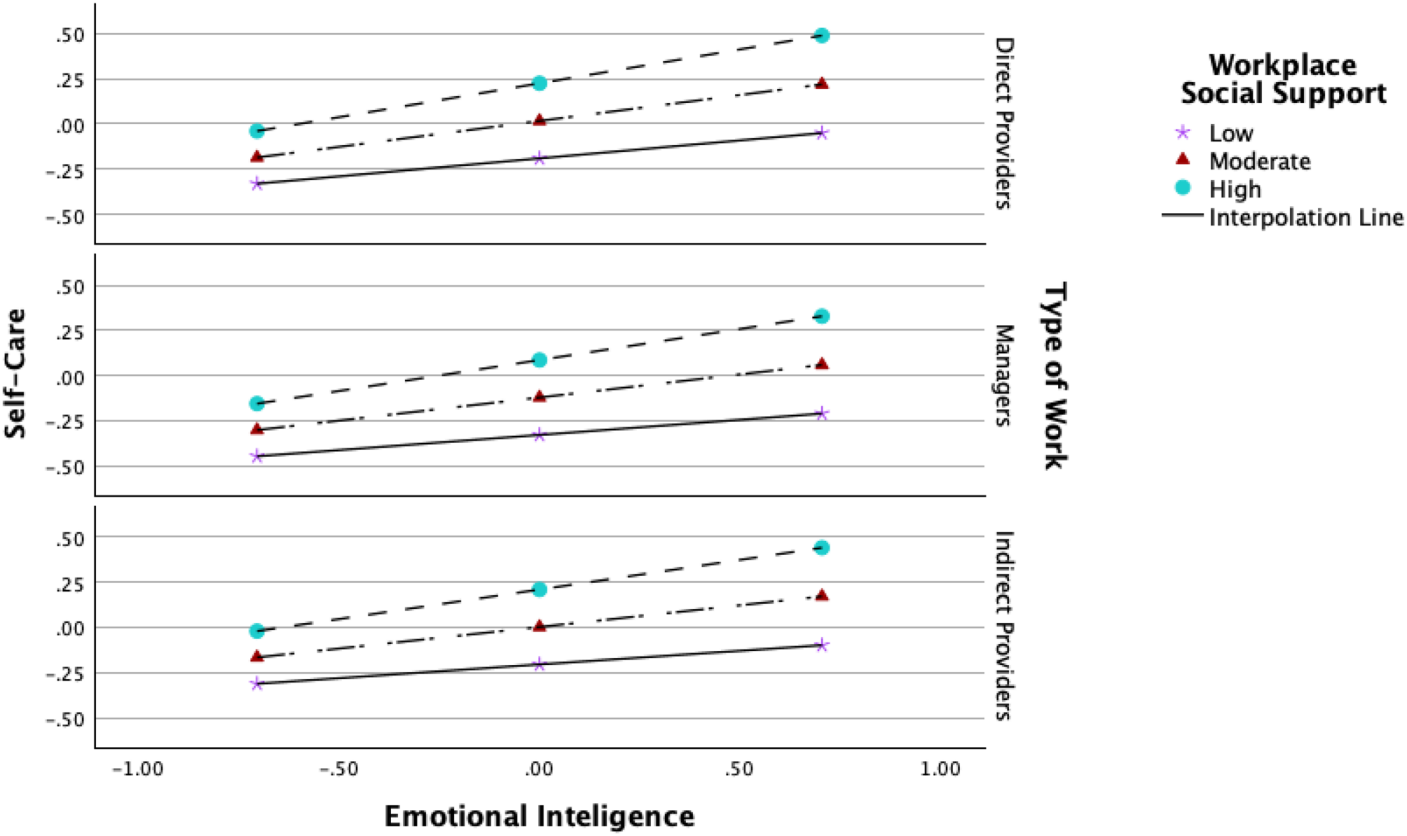
Conditional effects of EI on self-care. Fig. 5 shows the visual representation of the conditional effects of workplace social support on the relationship of emotional intelligence and self-care for direct providers, non-direct providers, and managers. Johnson-Neyman simple slopes analysis was conducted using Andrew Hayes’ PROCESS macro [68] to further explore the moderating effect of work on the relationship between emotional intelligence and self-care. Increasing levels of workplace social support resulted in increased positive effects for all types of providers (β = 0.106, p < 0.001)

The conditional effects of emotional intelligence on self-care were significant for workplace social support (R = 0.27, *t* = 12.533, *p* < 0.001)(Table 11). Workplace social support positively predicted self-care (β = 0.250, *p* < 0.001). Type of work predicted self-care effects, with a significant decrease for non-direct providers compared to direct providers(β = 0.137, *p* = 0.021) (Table 13 and Fig. 5).

##### Conditional effects of self-care on outcomes

The conditional effects of self-care on ProQOL outcomes were not significant. Workplace social support—but not type of work—significantly predicted ProQOL outcomes: positively for compassion satisfaction (β = 0.257, *p* < 0.001), negatively for burnout (β = −0.357, *p* < 0.001), and compassion fatigue (β = −0.272, *p* < 0.001) (Table 11). Type of work modulated the effect of social support on compassion satisfaction for non-direct providers (β = −0.303, *p* = 0.02). Some conditional effects were non-significant, for example, under conditions of low workplace social support for managers. Non-significant effects were retained to ensure full transparency and accuracy in reporting, consistent with statistical best practices in moderated mediation research [71] since they delineate the contextual boundaries of the model’s applicability.

**Table 13:** Conditional effects of EI on self-care at values of the moderator

Conditional effects EI on SC				B	SE	t	p
Emotional intelligence (EI)				0.286	0.051	5.624	0.000
Workplace social support (WPSS)				0.250***	0.030	8.452	0.000
Interaction EI*WPSS				0.106***	0.030	3.540	0.000
Z1 (Transition DP to MN)				−0.014	0.060	−0.236	0.814
Z2 (Transition DP to NDP)				−0.137*	0.059	−2.326	0.021
Interaction EI*Z1				−0.047	0.092	−0.515	0.607
Interaction EI*Z2				−0.032	0.087	−0.361	0.719
Gender				0.031	0.061	0.500	0.617
Age				−0.001	0.002	−0.294	0.769
Unconditional interactions				*R*^2^	F	df	p
Interaction EI*WPSS				0.027***	12.533	(1;296)	0.000
Interaction EI*TOW				0.001	0.154	(2;296)	0.857
Interaction BOTH (EI)				0.029**	4.485	(3,296)	0.004
Conditional effects EI on SC at values of the moderators							
	Workplace Social Support	Type of work		B	SE	t	p
	−0.830	Direct providers		0.199***	0.043	4.587	0.000
		Managers		0.151	0.082	1.846	0.066
		Non direct providers		0.167*	0.077	2.167	0.031
	0.000	Direct providers		0.286***	0.051	5.624	0.000
		Managers		0.239**	0.077	3.087	0.002
		Non direct providers		0.255***	0.072	3.527	0.000
	0.830	Direct providers		0.374***	0.067	5.552	0.000
		Managers		0.327***	0.081	4.048	0.000
		Non direct providers		0.343***	0.076	4.524	0.000

*n* = 306. This table shows the conditional effects of EI on self-care at various levels of workplace social support and type of work (Path a). Note that the conditional effects of type of work were reported as non-significant, yet significantly increased the effects, albeit marginal, when combined with workplace support (R2 = 0.29, *t* = 4.485, *p* = 0.004). Probing the interaction at the values of the moderators demonstrated that the conditional effects were not significant in all situations, for example, when support is low for managers. * *p* < 0.05. **p < 0.01.***p < 0.001

**Table 14:** Conditional direct effects at values of the moderator

Conditional Direct Effects on Outcomes at Values of the Moderators				
Workplace social support	Type of work	Burnout	Compassion fatigue	Compassion satisfaction
−0.830	Direct providers	−0.183*	−0.229*	0.161*
	Managers	−0.054	−0.306	0.031
	Non-direct providers	0.302	0.047	−0.196
0.000	Direct providers	−0.151	−0.203	0.228*
	Managers	−0.021	−0.280	0.098
	Non-direct providers	0.334*	0.072	−0.129
0.830	Direct providers	−0.118	−0.177	0.294*
	Managers	0.011	−0.254	0.164
	Non-direct providers	0.367*	0.098	−0.063

Table 14 shows the conditional direct effects at values of workplace social support and type of work. Note that the effects were not significant at specific values of the moderators, for example, on compassion fatigue when the support was high for direct providers. N = 306. Level of confidence (CI) 95.0000, number of bootstrap samples 5000. * CI does not overlap with zero

#### Conditional direct effects of EI on ProQOL

**Fig. 6:**
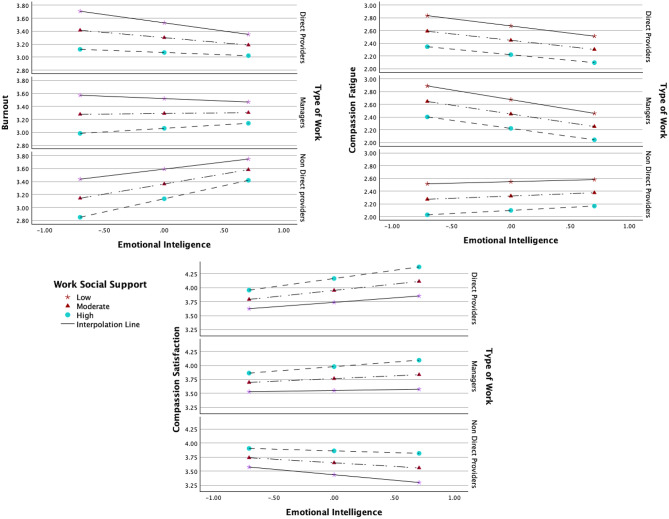
Conditional effects of EI on ProQOL. Fig. 6 shows the visual representation of the conditional effects of workplace social support on the direct and indirect relationships between emotional intelligence and the three components of ProQOL for direct providers, non-direct providers, and managers, using a Johnson-Neyman simple slopes analysis conducted using Andrew Hayes’ PROCESS macro (Hayes, [68]). Increasing the levels of workplace social support resulted in decreased, compassion fatigue, and in increased compassion satisfaction for all types of providers with: direct-providers with the same levels of emotional intelligence (β = 0.106, p < 0.001). However, when the levels of emotional intelligence increased, the conditional effects slightly decreased and even inverted for managers for burnout. They changed direction on non-direct providers for all outcomes, regardless of the type of social support (non-ordinal relationships). This interaction is considered disordinal

**Fig. 7:**
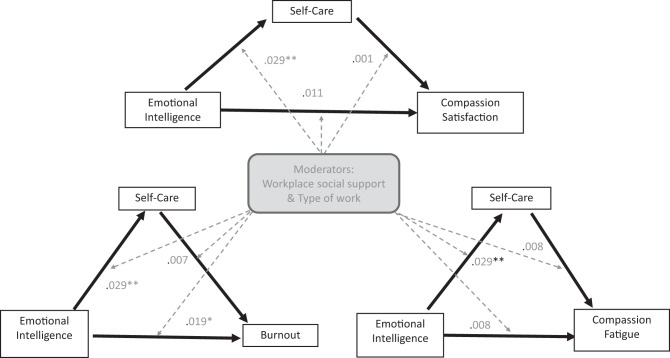
Interactions coefficients of the moderators. *N* = 324. Figure 7 shows the interaction coefficients of the effects in the moderated mediation analysis. Arrows indicate directional effects: straight arrows represent direct or indirect (mediated) effects via self-care, while dashed arrows denote interaction effects (moderation). While not all the interaction coefficients were significant, probing interactions at different levels of support showed significant conditional direct and indirect effects on all outcomes. p** < 0.01, p* < 0.05

The conditional direct effects of emotional intelligence on the dependent variables (path c’) were significant for both moderators on burnout (R2 = 0.019, *t* = 2.847, *p* = 0.038), and for different work groups on burnout (β = 0.485, *p* = 0.015) and compassion satisfaction (β = −0.357, *p* = 0.035). While not all interaction coefficients were significant, probing conditional effects across different workplace social support levels revealed significant relationships influencing ProQOL components (Table 14). Furthermore, Fig. 6 shows that when emotional intelligence increased, the transition from direct to indirect providers inverted the direction of the conditional effects of workplace social support on the relationship between emotional intelligence and burnout and compassion fatigue (from negative to positive), and between emotional intelligence and compassion satisfaction (from positive to negative). A summary of all interaction coefficients is given in Fig. 7.

### Results summary

In sum, emotional intelligence, self-care, and workplace social support were positively associated with compassion satisfaction and negatively with burnout and compassion fatigue. Self-care mediated the effect of EI on all ProQOL components, with higher mediation effects observed in professional self-care aspects.

The direct effect of emotional intelligence was significant on compassion fatigue, on burnout only within the emotion regulation component, and on compassion satisfaction only within emotion utilisation. Moderated mediation analyses showed that both workplace social support and type of work moderated the direct and indirect effects of EI. However, increasing emotional intelligence of non-direct providers, for certain types of support—particularly supervisor support and sense of community—decreased and reversed the conditional effects of workplace social support on burnout and compassion satisfaction.

## Discussion

### Protective effects on professional quality of life

This study examined relationships between professional quality of life (ProQOL) and variables including emotional intelligence, self-care, workplace social support, and type of work among healthcare workers.

In line with Hypothesis 1, findings revealed significant relationships among these variables, indicating emotional intelligence, self-care, and workplace social support mutually exert protective effects on each other and on ProQOL. These results are consistent with previous research demonstrating similar protective relationships [19, 44].

Significant differences emerged between healthcare worker groups. Direct providers reported notably lower workplace social support and higher compassion fatigue compared to non-direct providers and managers, resonating with research identifying direct providers as more vulnerable to compassion fatigue [49, 73]. Furthermore, workplace social support was a salient mediating factor influencing compassion fatigue across all worker groups, consistent with findings that underscore risks to compassion fatigue beyond direct-providers such as non-direct providers [48], customer contact workers [74], and educators [75]. Low workplace social support has been robustly linked to elevated compassion fatigue [76], while increasing social and emotional support effectively reduces compassion fatigue among direct providers [10].

The observation that managers scored higher in emotional intelligence aligns with studies relating managerial roles and hospital size to elevated emotional intelligence levels [77]. This may reflect recruitment and training processes that favour and develop these skills in leadership positions [78], although comparative studies between healthcare providers and managers remain sparse. The sample’s mean emotional intelligence levels surpassed those reported in prior general population and healthcare professional studies [79–82]. This observation contrasts expectations rooted in cultural differences, such as Australia’s individualistic orientation compared to more collectivist cultures [83, 84]. but may be explained by longitudinal increases in emotional intelligence over time [85].

Unexpectedly, no significant differences in burnout or compassion satisfaction were found between worker groups. This may relate to comparatively higher burnout and lower compassion satisfaction scores in this sample relative to other countries [18, 20], possibly compounded by attitude and behavioural shifts associated with the COVID-19 pandemic [86–88]. Additionally, self-care scores did not differ significantly across groups, with overall self-care engagement being lower than in previous studies [63, 66], potentially reflecting post-pandemic effects and regional transmission of attitudes toward self-care [89].

Limited associations among covariates were observed, notably emotional intelligence relating to gender and supervisor support to age, paralleling prior research linking these differences to work experience and cultural conceptualisations of gender roles [44, 55].

### The mediating effects of self-care and its domains

Consistent with Hypothesis 2, emotional intelligence influenced both perception and practice components of self-care, which in turn affected professional quality of life. Although prior studies showed self-care mediating the impact of emotional intelligence on perceived stress [44], this study uniquely tested self-care domains (perception and practice, personal and professional) as mediators between emotional intelligence and ProQOL.

While self-care exerted significant mediating effects, the relative effect magnitudes of personal self-care perception and practice on ProQOL were marginal and low compared to professional self-care perception and practice. These results align with prior work contrasting programmatic perceptions of self-care with reported use of strategies concerning quality of life [90]. Additional qualitative evidence suggests that personal self-care alone is insufficient to manage work-related stress effectively, emphasising the importance of professional self-care initiatives [91].

### Emotional intelligence and its pathways to outcomes

Findings relevant to Hypothesis 2 also indicated that emotional intelligence influenced compassion satisfaction and burnout primarily through indirect pathways mediated by self-care, whereas its effect on compassion fatigue operated via both indirect and direct pathways. Thus, emotional intelligence impacts different ProQOL components through distinct mechanisms. Other studies support the role of various mediators and moderators, such as effective coping strategies [92]) and empathy [93], which may be modulating emotional intelligence’s direct effects on compassion fatigue.

Moreover, components of emotional intelligence exert differential direct influences. Only the “use of emotions” component was significant for burnout, which aligns with findings of its significant indirect effects on work performance via burnout [94]. Meanwhile, other evidence suggests that modulators, such as leader support, may be affecting the unexpected non-significant effects of self-emotion appraisal on burnout [94]. The significant effects of all components but “other-emotional appraisal” on compassion fatigue are supported by evidence that self-emotional appraisal relates significantly to job satisfaction mediated by surface-acting [95]-which is linked to compassion fatigue [96]. The relevance of “regulation of emotions” for compassion satisfaction might be related to the effects on emotional regulation on reducing compassion fatigue moderated by cognitive empathy [93]. Finally, the non-significant effects of other-oriented emotional appraisal component on all ProQOl components are supported by evidence that self-focused emotional intelligence components mitigate stress and ill-health, while other-oriented components contribute more to social and work success [97, 98].

### Influencing effects and the role of moderators

Supporting Hypothesis 3, workplace social support and type of work significantly moderated both the direct and indirect effects of emotional intelligence on ProQOL components. The moderation effects were stronger on the influence of emotional intelligence on self-care than on the impact of self-care on ProQOL, particularly among direct providers. This aligns with Ma et al.’s [72] findings that workplace environment factors, such as ostracism, attenuate the enactment of emotional intelligence skills despite high individual scores, highlighting the necessity of supportive work environments for individuals to leverage their resources effectively.

A novel contribution of this study is demonstrating that varying levels of workplace social support can differentially influence the relationship between the two predictors (EI and self-care) and ProQOL outcomes according to the provider type for increasing levels of emotional intelligence. Workplace social support, particularly supervisor support, enhanced the positive effects of emotional intelligence on ProQOL for direct providers and in most circunstances for managers but paradoxically reversed these effects for non-direct providers, increasing burnout and compassion fatigue and decreasing compassion satisfaction.

A potential explanation for these findings is the elevated burnout levels observed in the sample. Research indicates that a heightened sense of community in stressful environments can augment perceived work demands for support, thereby exacerbating burnout [99]. Also, support has been shown to have negative effects when supervisors exhibit simultaneous undermining and supportive behaviours [100], workaholism [101], or are deficient in coaching abilities, especially regarding emotional management [102]. The findings echo evidence that, depending on context, extremely low or high levels of supervisor support can precipitate burnout [103, 104], suggesting the importance of tailored supervisor support contingent on provider roles and emotional regulation abilities.

### Limitations, implications, and conclusion

While this study offers valuable insights, several limitations should be acknowledged. Reliance on self‑report measures raises the possibility of bias, and future studies could improve measurement validity by incorporating culturally adjusted items [101, 105] or objective indicators such as physiological stress markers. The broad categorisation of professional roles restricted understanding of task‑specific impacts, and greater role granularity—including shared or overlapping responsibilities—could better illuminate workload distribution and emotional labour dynamics.

The cross‑sectional design precludes causal inference, and in line with Bronfenbrenner’s ecological framework [34], which emphasises the interplay of layered systemic influences, many relevant factors and their interactions could not be captured [106]. Furthermore, findings are drawn from a single Local Health District in NSW, comprising a predominantly female, mid‑career workforce with explicit collaborative values [60, 107] which may limit generalisability and replication in settings with different workforce compositions and cultural climates. Replication in diverse healthcare contexts is recommended to strengthen external validity.

Despite these constraints, the study provides a nuanced understanding of how and when individual capacities and organisational resources shape professional quality of life (ProQOL) in healthcare settings. Professional self‑care emerged as a particularly important protective factor, exerting stronger influence on mitigating work‑related stress than personal self‑care approaches. This reinforces the need for workplace-embedded strategies that actively promote sustainable, role-specific self-care practices. Future research should explore the interplay between personal and professional self‑care, and develop integrative theoretical and operational frameworks.

Emotional intelligence also had a significant positive impact on all three ProQOL components, operating both directly and indirectly through self‑care. Different emotional intelligence components shaped outcomes in distinct ways: “self‑focus” emotional intelligence components were linked with higher compassion fatigue, the “use of emotions” with greater compassion satisfaction, and “regulation of emotions” with reduced burnout. These patterns highlight the need for finely tuned training interventions that prioritise the most relevant emotional capacities for specific roles.

Workplace social support and type of work further moderated these relationships, with stronger protective effects evident among direct care providers. For non‑direct providers, however, higher emotional intelligence was associated with attenuated—or even reversed—effects on burnout, compassion fatigue, and compassion satisfaction, suggesting that contextual fit can alter the role of personal resources.

In practical terms, fostering a culture that normalises and supports professional self‑care, investing in emotionally skilled leadership and supervision, and providing responsive workplace supports workplace social support—particularly for frontline providers—are key strategies for safeguarding ProQOL. Qualitative, action research, and serial/parallel mediation methods may be beneficial for unpacking complex causal pathways. By aligning workforce capabilities with a context‑responsive support environment, healthcare organisations can better promote wellbeing, resilience, and professional satisfaction among their staff.

## Electronic supplementary material

Below is the link to the electronic supplementary material.

Supplementary Material 1

Supplementary Material 2

Supplementary Material 3

## Data Availability

The data that support the findings of this study are available from Brunel University London, but restrictions apply to the availability of these data, which were used under authorisation for the current study, and so are not publicly available. Data are however available from the authors upon reasonable request and with permission of Central Coast Local Health District and Brunel University London.

## Footnotes

1 Parameters selected in the software included mean centre-only continuous variables for both models and conditioning values (−1SD, Mean, +1SD) for model 76 only.

## Abbreviations

BET: Bronfenbrenner Ecological Theory
CCLHD: Central Coast Local Health District
CFM: The Compassion Fatigue Model
JDR: The Job Demands-Resources Theory
ProQOL: Professional Quality of Life
