# Supplementary material for: Professional quality of life is related to emotional intelligence, self-care, and work conditions in healthcare workers: findings from a moderated mediation analysis
Source: BMC Health Serv Res. 2025 Oct 21;25:1381. doi: 10.1186/s12913-025-13437-7 (PMC12539004; doi:10.1186/s12913-025-13437-7)
Supplement: Supplementary file 1 — Supplementary Material 1 [file 12913_2025_13437_MOESM1_ESM.pdf]

Supplement A - Figures showing means distribution of scales' items

Figure A.1 *Means Distribution For Items From The Short Professional Quality Of Life Scale[a].*

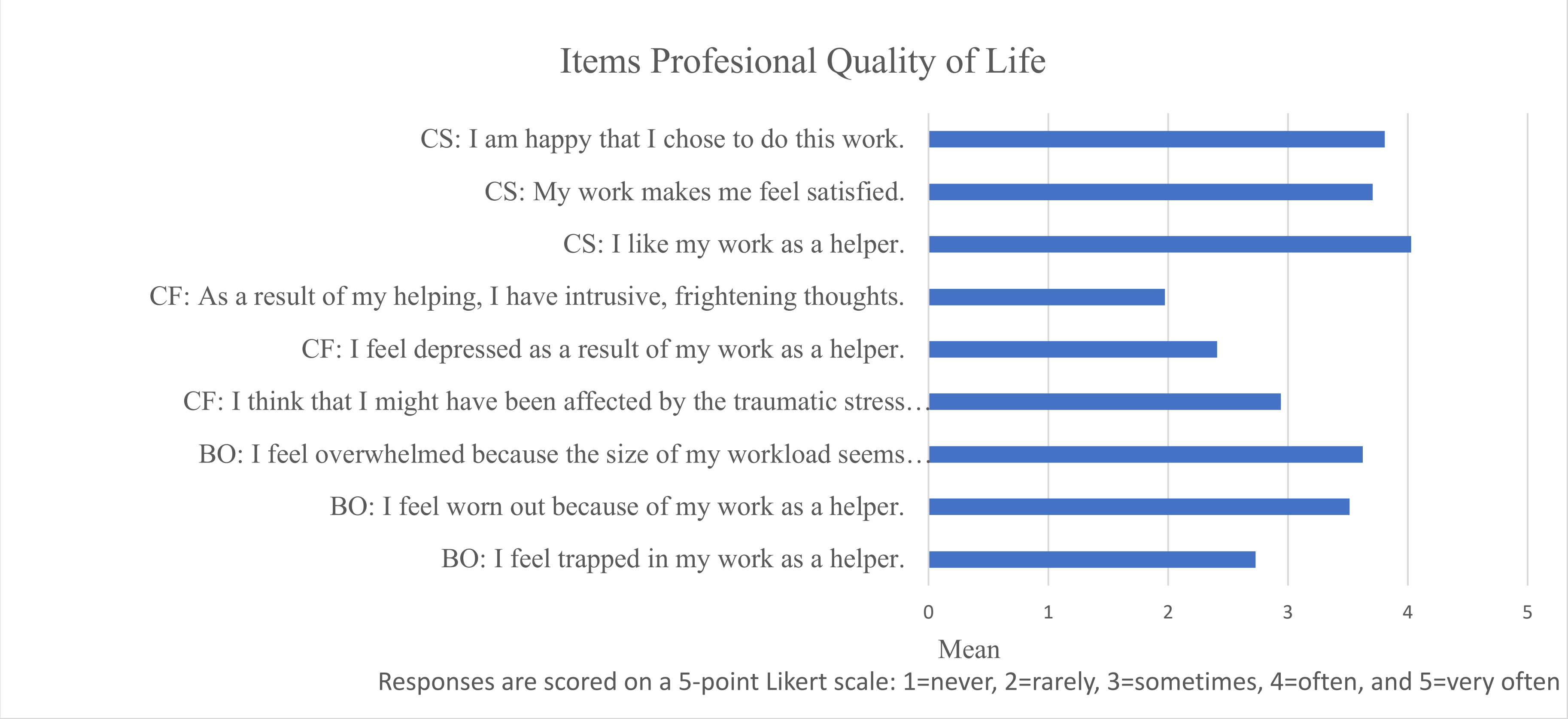

Legend: Scale from *Galiana, 2022.* N 343. CS for Compassion Satisfaction, CF for Compassion Fatigue (CF), and BO for Burnout.

Figure A.2 *Mean distribution for items from the Wong and Law Emotional Intelligence Scale*[a]

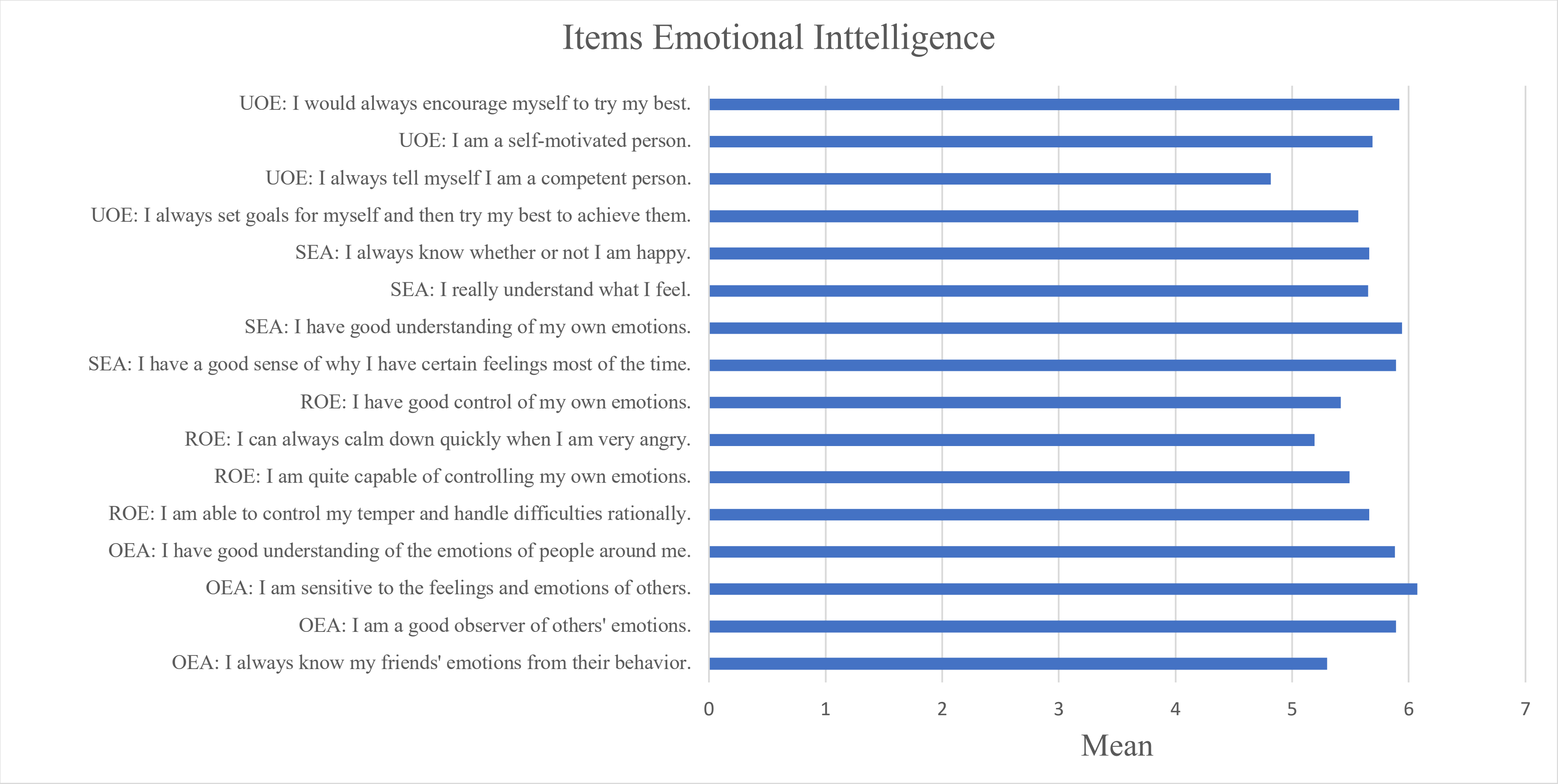

Legend: [a]*Items in scale from Wong & Law, 200).*N 343. Responses are scored on a 7-point Likert scale: 1=strongly disagree, 2=disagree, 3=slightly disagree, 4=neither agree nor disagree, 5=slightly agree, 6=agree, and 7=strongly agree. UOE for use of emotions, SEA for self-emotions appraisal, ROE for regulation of emotions, and OEA for other's emotions appraisal.

Figure A.3 *Mean distribution for items Self-Care Practice (adapted from SCP (Lee et al., 2020) and MSCS (Cook-Cottone & Guyker, 2018).*

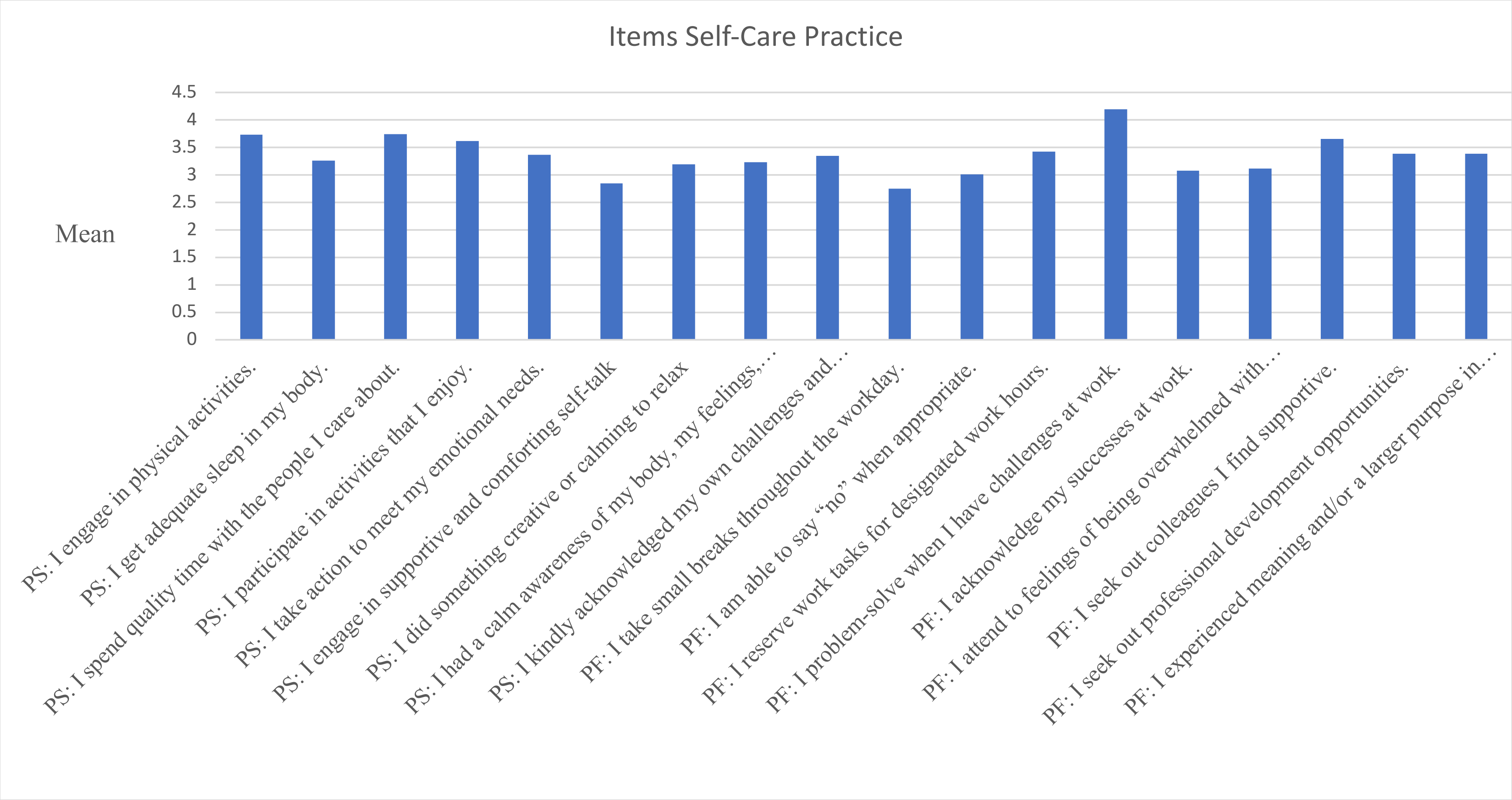

Legend: N 343. Responses are scored on a five-point Likert scale with 1= never, 2=rarely, 3=sometimes, 4=often and 5=very often. PS for personal practice and PF for professional practice.

Figure A.4 *Means items from the Self-Care Perception Scale (Bloomquist et al, 2015).*

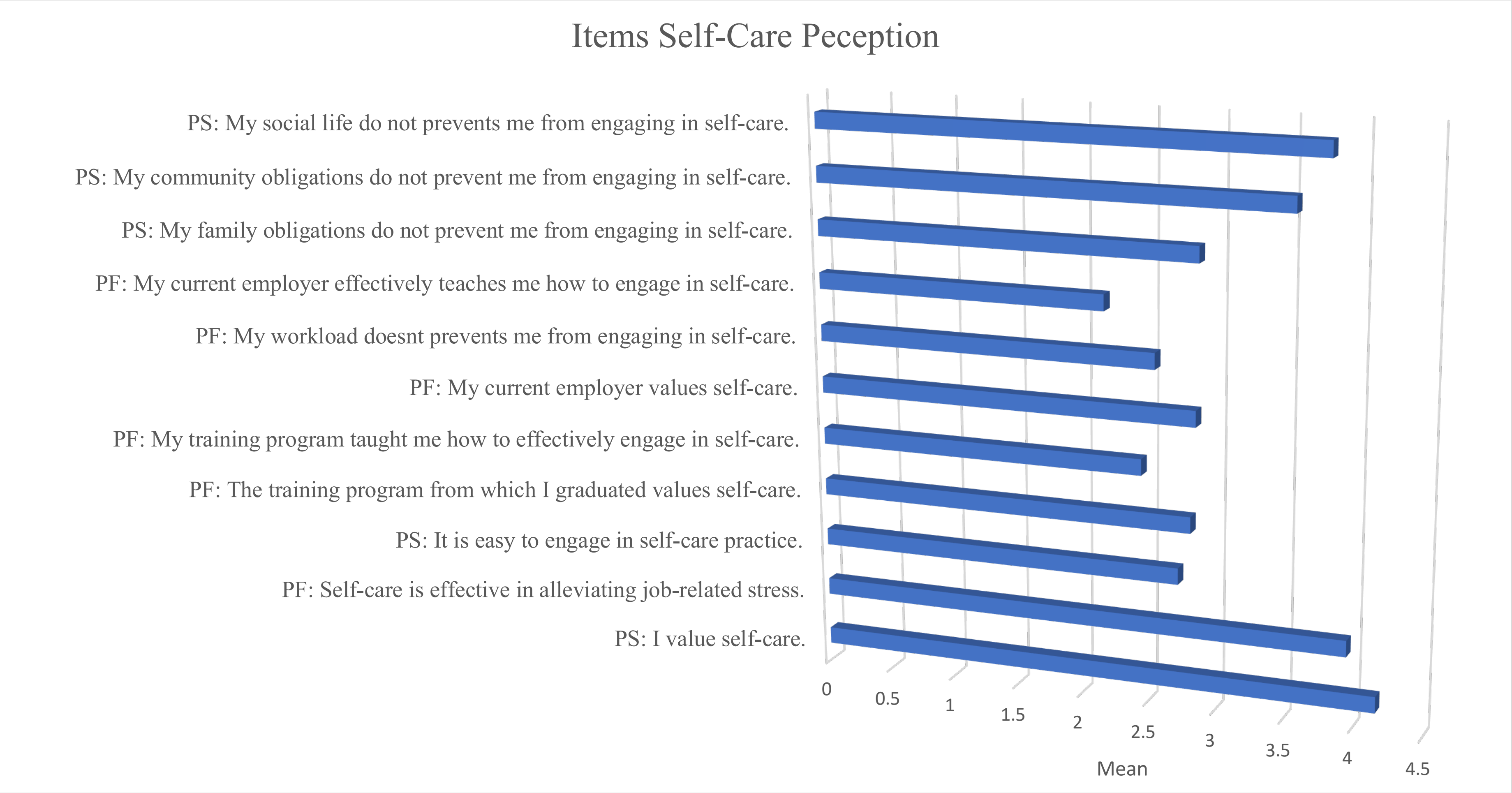

Legend: N 343. Responses are scored on a five-point Likert scale with 1= never, 2=rarely, 3=sometimes, 4=often and 5=very often. PS for personal practice and PF for professional practice.

Figure A.5 *Means items for Workplace Social Support* scale (adapted from COPSOQ III).

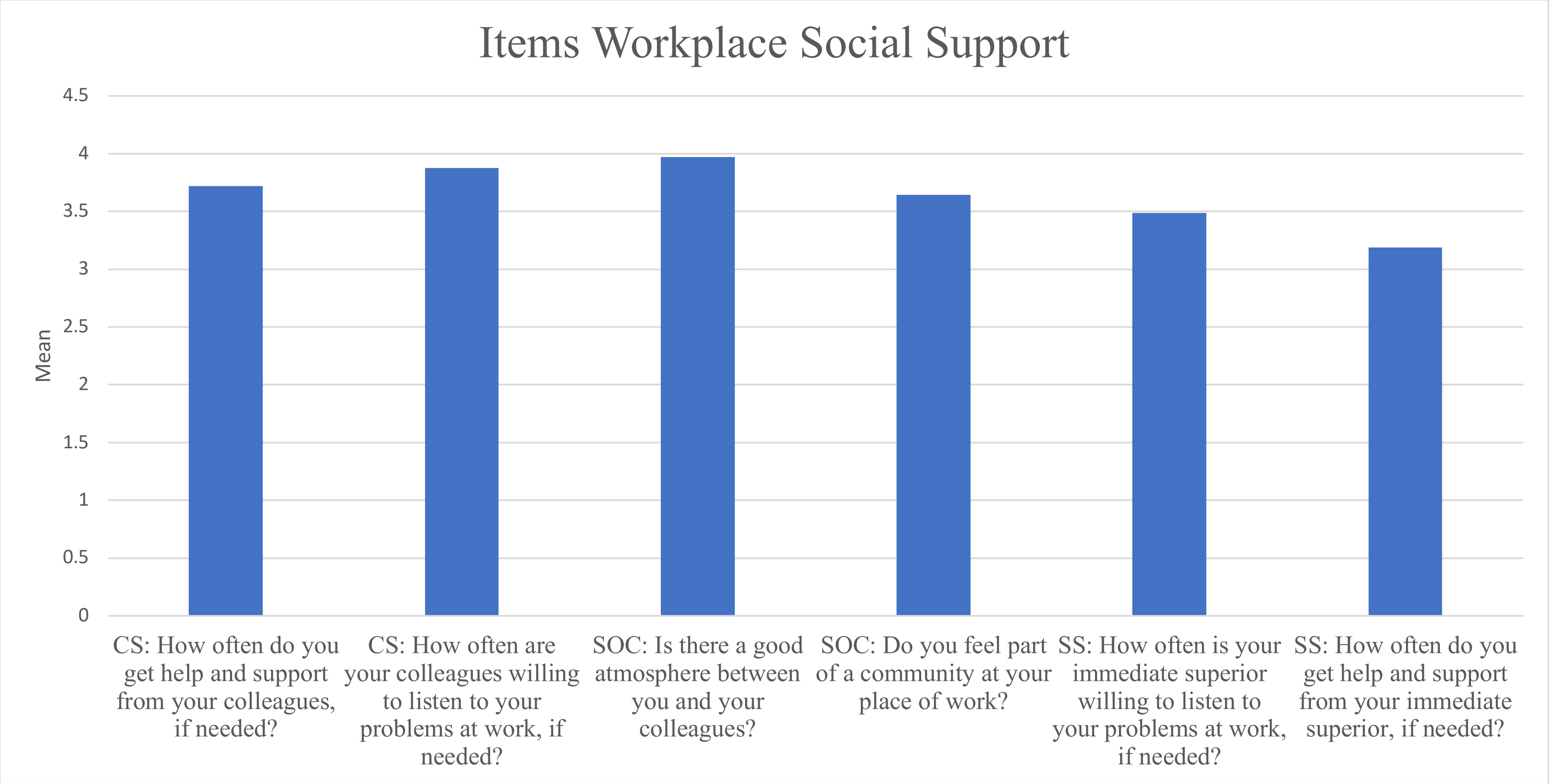

Legend: N 343. Responses scored on a 5-point Likert type scale: 1=Never/hardly ever, 2=seldom, 3=sometimes, 4=often, and 5=strongly always. SOC for sense of community, SS for supervisor support, and CS for colleague support.
