# Supplementary material for: Professional quality of life is related to emotional intelligence, self-care, and work conditions in healthcare workers: findings from a moderated mediation analysis
Source: BMC Health Serv Res. 2025 Oct 21;25:1381. doi: 10.1186/s12913-025-13437-7 (PMC12539004; doi:10.1186/s12913-025-13437-7)
Supplement: Supplementary file 2 — Supplementary Material 2 [file 12913_2025_13437_MOESM2_ESM.pdf]

Supplement B Results mediation analysis with sub-scales

Table B.1 Results Mediation Analysis with Subscales for Burnout

Burnout (DV)	Emotional Intelligence and its components (IV)				
	EI	SEA	OEA	ROE	UOE
**Self-care (M)**					
Effect IV on M (a)	.316***	.223***	.091*	.135***	.206***
Effect of M on DV (b)	-.876***	-.879***	-.950***	-.879***	-.924***
Direct effect (c’)	-.105	-.092	.033	-.090*	-.021
Indirect effect (a*b)	-.277 CI*	-.196 CI*	-.086 CI*	-.118 CI*	-.191 CI*
Total effects (c)	-.382***	-.287***	-.053	-.208***	-.212***
Controlled variables on M/DV	No sig	No sig	No sig	No sig	No sig
**Self-care perception (M)**					
Effect IV on M (a)	.235***	.185***	.056	.117***	.127***
Effect of M on DV (b)	-.808***	-.817***	-.879***	-.825***	-.833***
Direct effect (c’)	-.192**	-.136*	-.004	-.112**	-.106*
Indirect effect (a*b)	-.190 CI*	-.151 CI*	-.049	-0.96 CI*	-.106 CI*
Total effects (c)	-.382***	-.287***	-.053	-.208***	-.212***
Controlled variables on M/DV	DV: G2, -.238*	DV: G2, -.242*	No sig	No sig	No sig
**Self-care perception personal**					
Effect IV on M (a)	.151**	.150***	.026	.076*	.063
Effect of M on DV (b)	-.419***	-412***	-.479***	-.436***	-.448***
Direct effect (c’)	-.319***	-.225***	-.041	-.175***	-.183***
Indirect effect (a*b)	-.063 CI*	-.062 CI*	-.012	-.033 CI*	-.028
Total effects (c)	-382***	-.287***	-.053	-208***	-.212***
Controlled variables on M/DV	No sig	No sig	No sig	No sig	No sig
**Self-care perception professional (M)**					
Effect IV on M (a)	.306***	.215***	.081	.151***	.180***
Effect of M on DV (b)	-.609***	-.617***	-.666***	-.623***	-.630***
Direct effect (c’)	-.195**	-.155**	.002	-.114**	-.098*
Indirect effect (a*b)	-.187 CI*	-.133 CI*	-.054	-.094 CI*	-.113 CI*
Total effects (c)	-.382***	-.287***	-.053	-.208***	-.212***
Controlled variables on M/DV	DV: G2, .265*	DV: G2, .278*	No sig	No sig	DV: G2, .219*
**Self-care practice (M)**					
Effect IV on M (a)	.356***	.241***	.108**	.143***	.246***
Effect of M on DV (b)	-.596***	-.608***	-.699***	-.622***	-649***
Direct effect (c’)	-.169*	-.141*	.023	-.119**	-0.52
Indirect effect (a*b)	-.213 CI*	-.147 CI*	-.075 CI*	-.089 CI*	-.160 CI*
Total effects (c)	-382***	-.287***	-.053	-.208***	-.212***
Controlled variables on M/DV	No sig	No sig	No sig	No sig	No sig
**Self-care practice personal (M)**					
Effect IV on M (a)	.422***	.284***	.118*	.186***	.278***
Effect of M on DV (b)	-.314***	-.334***	-.003	-.351***	-.351***
Direct effect (c’)	-.250**	-.192**	-.422***	-.143**	-.114*
Indirect effect (a*b)	-.132 CI*	-.095 CI*	-.050 CI*	-.065 CI*	-.098 CI*
Total effects (c)	-.382***	-.287***	-.053	-.208***	-.107***
Controlled variables on M/DV	No sig	No sig	No sig	No sig	No sig
**Self-care practice professional (M)**					
Effect IV on M (a)	.291***	.198***	.098*	.101***	.214***
Effect of M on DV (b)	-.588***	-602***	-.684***	-.623***	-.626***
Direct effect (c’)	-.211**	-168**	.015	-.145**	-0.78
Indirect effect (a*b)	-.171 CI*	-.119 CI*	-.067 CI*	-.063 CI*	-.134 CI*
Total effects (c)	-382***	-.287***	-.053	-.208***	-.212***
Controlled variables on M/DV	No sig	No sig	No sig	No sig	No sig

Legend: N 324, sig at *** p < .000, Sig at ** p < .01, sig at * p < .05, sig CI* do not include zero,, confidence intervals 95, and number of bootstraps samples 5000.

Table B.2 Results Mediation Analysis with Subscales for Compassion Fatigue

**Compassion Fatigue (DV)**	**Emotional Intelligence and its components (IV)**				
	**EI**	**SEA**	**OEA**	**ROE**	**UOE**
**Self-care (M)**					
Effect IV on M (a)	.316***	.223***	.091*	.135***	.206***
Effect of M on DV (b)	-.619***	-.663***	-.751***	-.672***	-.666***
Direct effect (c’)	-.222***	-.138*	-.040	-.124**	-.103*
Indirect effect (a*b)	-.196*	-.148 CI*	-.068 CI*	-.090 CI*	-.137 CI*
Total effects (c)	-.418***	-.286***	-.108	-.214***	-.241***
Controlled variables on M/DV	No sig	No sig	No sig	No sig	No sig
**Self-care perception (M)**					
Effect IV on M (a)	.235***	.185***	0.56	.117***	.127***
Effect of M on DV (b)	-.596***	-.621***	-.692***	-.631***	-.628***
Direct effect (c’)	-.278***	-.171**	-.069	-.140**	-.161***
Indirect effect (a*b)	-.140 CI*	-.115 CI*	-.039	-.074 CI*	-.080 CI*
Total effects (c)	-.418***	-.286***	-.108	-.214***	-.241***
Controlled variables on M/DV	No sig	No sig	No sig	No sig	No sig
**Self-care perception personal (M)**					
Effect IV on M (a)	.151**	.150***	.026	.076*	.063
Effect of M on DV (b)	-.294***	-.293***	-.362***	-.318***	-.327***
Direct effect (c’)	-.374***	-.242***	-.098	-.190***	-.220***
Indirect effect (a*b)	-.044 CI*	-.044 CI*	-.009	-.024 CI*	-.021
Total effects (c)	-.418***	-.286***	-.108	-.214***	-.241***
Controlled variables on M/DV	No sig	No sig	No sig	No sig	No sig
**Self-care perception professional (M)**					
Effect IV on M (a)	.306***	.215***	.081	.151***	.180***
Effect of M on DV (b)	-.459***	-.482***	-.533***	-.487***	-.482***
Direct effect (c’)	-.277***	-.182**	-.064	-.141**	-.154**
Indirect effect (a*b)	-.141 CI*	-.103 CI*	-.043	-.073 CI*	-.087 CI*
Total effects (c)	-.418***	-.286***	-.108	-.214***	-.241***
Controlled variables on M/DV	No sig	No sig	No sig	No sig	M: G2, -.219*
**Self-care practice (M)**					
Effect IV on M (a)	.356***	.241***	.108**	.143***	.246***
Effect of M on DV (b)	-.406***	-.457***	-.554***	-.475***	-.451***
Direct effect (c’)	-.273***	-.175**	-.048	-.146**	-.130*
Indirect effect (a*b)	-.145 CI*	-.110 CI*	-.060 CI*	-.068 CI*	-.111 CI*
Total effects (c)	-.418***	-.286***	-.108	-.214***	-241***
Controlled variables on M/DV	No sig	No sig	No sig	No sig	No sig
**Self-care practice personal**					
Effect IV on M (a)	.422***	.284***	.118*	.186***	.278***
Effect of M on DV (b)	-.177*	-.224**	-.316***	-.241**	-.212**
Direct effect (c’)	-.343***	-.222***	-.070	-.169***	-.182***
Indirect effect (a*b)	-.075 CI*	-.064 CI*	-.037 CI*	-.045 CI*	-.059 CI*
Total effects (c)	-.418***	-.286***	-.108	-.214***	-.241***
Controlled variables on M/DV	No sig	No sig	No sig	No sig	No sig
**Self-care practice professional**					
Effect IV on M (a)	.291***	.198***	.098*	.101***	.214***
Effect of M on DV (b)	-.452***	-.489***	-.569***	-.513***	-.481***
Direct effect (c’)	-.286***	-.189**	-.052	-.162***	-.138**
Indirect effect (a*b)	-.132 CI*	-.097 CI*	-.056 CI*	-.052 CI*	-.103 CI*
Total effects (c)	-.418***	-.286***	-.108	-.214***	-.241***
Controlled variables on M/DV	No sig	No sig	No sig	No sig	No sig

Legend: N 324, sig at *** $p < .000$, Sig at ** $p < .01$, sig at * $p < .05$, sig CI* do not include zero, confidence intervals 95, and number of bootstraps samples 5000.

Table B.3 Results Mediation Analysis with Subscales for Compassion Satisfaction

**Compassion Satisfaction (DV)**	**Emotional Intelligence and its components (IV)**				
	**EI**	**SEA**	**OEA**	**ROE**	**UOE**
**Self-care (M)**					
Effect IV on M (a)	.316***	.223***	.91*	.135***	.206***
Effect of M on DV (b)	.748***	.797***	.822***	.775***	.740***
Direct effect (c’)	.118	.037	.004	.068	.092*
Indirect effect (a*b)	.236*	.178 CI*	.075 CI*	.104 CI*	.153 CI*
Total effects (c)	.355***	.214***	.078	.172***	.244***
Controlled variables on M/DV	No sig	No sig	No sig	No sig	No sig
**Self-care perception (M)**					
Effect IV on M (a)	.235***	.185***	.056	.117***	.127***
Effect of M on DV (b)	.543***	.578***	.623***	.576***	.551***
Direct effect (c’)	.227***	.107*	.043	.105**	.174***
Indirect effect (a*b)	.128 CI*	.107 CI*	.035	.067 CI*	.070 CI*
Total effects (c)	.355***	.214***	.078	.172***	.244***
Controlled variables on M/DV	No sig	No sig	No sig	No sig	No sig
**Self-care perception personal (M)**					
Effect IV on M (a)	.151**	.150***	.026	.076*	.063
Effect of M on DV (b)	.227**	.235**	.286***	.250***	.249***
Direct effect (c’)	.321***	.179***	.071	.154***	.229***
Indirect effect (a*b)	.034 CI*	.035 CI*	.007	.019 CI*	.016
Total effects (c)	.355***	.214***	.078	.172***	.244***
Controlled variables on M/DV	No sig	No sig	No sig	No sig	No sig
**Self-care perception professional (M)**					
Effect IV on M (a)	.306***	.215***	.081	.151***	.180***
Effect of M on DV (b)	.445***	.473***	.505***	.471***	.448***
Direct effect (c’)	.218***	.112*	.037	.101**	.164***
Indirect effect (a*b)	.136 CI*	.102 CI*	.041	.071 CI*	.081 CI*
Total effects (c)	.355***	.214***	.078	.172***	.244***
Controlled variables on M/DV	No sig	No sig	No sig	No sig	M: G2 -.219*
**Self-care practice (M)**					
Effect IV on M (a)	.356***	.241***	.108**	.143***	.246***
Effect of M on DV (b)	.595***	.641***	.676***	.627***	.592***
Direct effect (c’)	.143*	.059	.005	.083*	.099*
Indirect effect (a*b)	.212 CI*	.155 CI*	.073 CI*	.090 CI*	.145 CI*
Total effects (c)	.355***	.214***	.078	.172***	.244***
Controlled variables on M/DV	No sig	No sig	No sig	No sig	No sig
**Self-care practice personal**					
Effect IV on M (a)	.422***	.284***	.118*	.186***	.279***
Effect of M on DV (b)	.285***	.332***	.387***	.331***	.283***
Direct effect (c’)	.234***	.120*	.033	.111**	.165***
Indirect effect (a*b)	.120 CI*	.094 CI*	.045 CI*	.062 CI*	.079 CI*
Total effects (c)	.355***	.214***	.078	.172***	.244***
Controlled variables on M/DV	No sig	No sig	No sig	No sig	No sig
**Self-care practice professional**					
Effect IV on M (a)	.291***	.198***	.098*	.101***	.214***
Effect of M on DV (b)	.626***	.663***	.700***	.660***	.623***
Direct effect (c’)	.173**	.083	.009	.106**	.111**
Indirect effect (a*b)	.173 CI*	.132 CI*	.069 CI*	.067 CI*	.133 CI*
Total effects (c)	.355***	.214***	.078	.172***	.244***
Controlled variables on M/DV	No sig	No sig	No sig	No sig	No sig

Legend: N 324, sig at *** p < .000, Sig at ** p < .01, sig at * p < .05, sig CI* do not include zero, confidence intervals 95, and number of bootstraps samples 5000.
