# Supplementary material for: Professional quality of life is related to emotional intelligence, self-care, and work conditions in healthcare workers: findings from a moderated mediation analysis
Source: BMC Health Serv Res. 2025 Oct 21;25:1381. doi: 10.1186/s12913-025-13437-7 (PMC12539004; doi:10.1186/s12913-025-13437-7)
Supplement: Supplementary file 3 — Supplementary Material 3 [file 12913_2025_13437_MOESM3_ESM.docx]

| **Burnout (DV)** | **Emotional Intelligence and its components (IV)** | | | | |
| --- | --- | --- | --- | --- | --- |
|  | **EI** | **SEA** | **OEA** | **ROE** | **UOE** |
| **Self-care (M)** |  |  |  |  |  |
| Effect IV on M (a) | .316*** | .223*** | .091* | .135*** | .206*** |
| Effect of M on DV (b) | -.876*** | -.879*** | -.950*** | -.879*** | -.924*** |
| Direct effect (c’) | -.105 | -.092 | .033 | -.090* | -.021 |
| Indirect effect (a*b) | -.277 CI* | -.196 CI* | -.086 CI* | -.118 CI* | -.191 CI* |
| Total effects (c) | -.382*** | -.287*** | -.053 | -.208*** | -.212*** |
| Controlled variables on M/DV | No sig | No sig | No sig | No sig | No sig |
| **Self-care perception (M)** | | | | | |
| Effect IV on M (a) | .235*** | .185*** | .056 | .117*** | .127*** |
| Effect of M on DV (b) | -.808*** | -.817*** | -.879*** | -.825*** | -.833*** |
| Direct effect (c’) | -.192** | -.136* | -.004 | -.112** | -.106* |
| Indirect effect (a*b) | -.190 CI* | -.151 CI* | -.049 | -0.96 CI* | -.106 CI* |
| Total effects (c) | -.382*** | -.287*** | -.053 | -.208*** | -.212*** |
| Controlled variables on M/DV | DV: G2, -.238* | DV: G2, -.242* | No sig | No sig | No sig |
| **Self-care perception personal** | | | | | |
| Effect IV on M (a) | .151** | .150*** | .026 | .076* | .063 |
| Effect of M on DV (b) | -.419*** | -412*** | -.479*** | -.436*** | -.448*** |
| Direct effect (c’) | -.319*** | -.225*** | -.041 | -.175*** | -.183*** |
| Indirect effect (a*b) | -.063 CI* | -.062 CI* | -.012 | -.033 CI* | -.028 |
| Total effects (c) | -382*** | -.287*** | -.053 | -208*** | -.212*** |
| Controlled variables on M/DV | No sig | No sig | No sig | No sig | No sig |
| **Self-care perception professional (M)** | | | | | |
| Effect IV on M (a) | .306*** | .215*** | .081 | .151*** | .180*** |
| Effect of M on DV (b) | -.609*** | -.617*** | -.666*** | -.623*** | -.630*** |
| Direct effect (c’) | -.195** | -.155** | .002 | -.114** | -.098* |
| Indirect effect (a*b) | -.187 CI* | -.133 CI* | -.054 | -.094 CI* | -.113 CI* |
| Total effects (c) | -.382*** | -.287*** | -.053 | -.208*** | -.212*** |
| Controlled variables on M/DV | DV: G2, .265* | DV: G2, .278* | No sig | No sig | DV: G2, .219* |
| **Self-care practice (M)** |  |  |  |  |  |
| Effect IV on M (a) | .356*** | .241*** | .108** | .143*** | .246*** |
| Effect of M on DV (b) | -.596*** | -.608*** | -.699*** | -.622*** | -649*** |
| Direct effect (c’) | -.169* | -.141* | .023 | -.119** | -0.52 |
| Indirect effect (a*b) | -.213 CI* | -.147 CI* | -.075 CI* | -.089 CI* | -.160 CI* |
| Total effects (c) | -382*** | -.287*** | -.053 | -.208*** | -.212*** |
| Controlled variables on M/DV | No sig | No sig | No sig | No sig | No sig |
| **Self-care practice personal (M)** | | | | | |
| Effect IV on M (a) | .422*** | .284*** | .118* | .186*** | .278*** |
| Effect of M on DV (b) | -.314*** | -.334*** | -.003 | -.351*** | -.351*** |
| Direct effect (c’) | -.250** | -.192** | -.422*** | -.143** | -.114* |
| Indirect effect (a*b) | -.132 CI* | -.095 CI* | -.050 CI* | -.065 CI* | -.098 CI* |
| Total effects (c) | -.382*** | -.287*** | -.053 | -.208*** | -.107*** |
| Controlled variables on M/DV | No sig | No sig | No sig | No sig | No sig |
| **Self-care practice professional (M)** | | | | | |
| Effect IV on M (a) | .291*** | .198*** | .098* | .101*** | .214*** |
| Effect of M on DV (b) | -.588*** | -602*** | -.684*** | -.623*** | -.626*** |
| Direct effect (c’) | -.211** | -168** | .015 | -.145** | -0.78 |
| Indirect effect (a*b) | -.171 CI* | -.119 CI* | -.067 CI* | -.063 CI* | -.134 CI* |
| Total effects (c) | -382*** | -.287*** | -.053 | -.208*** | -.212*** |
| Controlled variables on M/DV | No sig | No sig | No sig | No sig | No sig |
